# Computational Modeling of Supramolecular Metallo-organic Cages–Challenges and Opportunities

**DOI:** 10.1021/acscatal.2c00837

**Published:** 2022-05-02

**Authors:** Tomasz K. Piskorz, Vicente Martí-Centelles, Tom A. Young, Paul J. Lusby, Fernanda Duarte

**Affiliations:** † Chemistry Research Laboratory, University of Oxford, Mansfield Road, Oxford OX1 3TA, United Kingdom; ‡ Instituto Interuniversitario de Investigación de Reconocimiento Molecular y Desarrollo Tecnológico (IDM), Universitat Politècnica de València, Universitat de València, Valencia 46022, Spain; § EaStCHEM School of Chemistry, University of Edinburgh, Joseph Black Building, David Brewster Road, Edinburgh, Scotland EH9 3FJ, United Kingdom

**Keywords:** supramolecular chemistry, metallo-organic cages, computational modeling, biomimetic catalysis

## Abstract

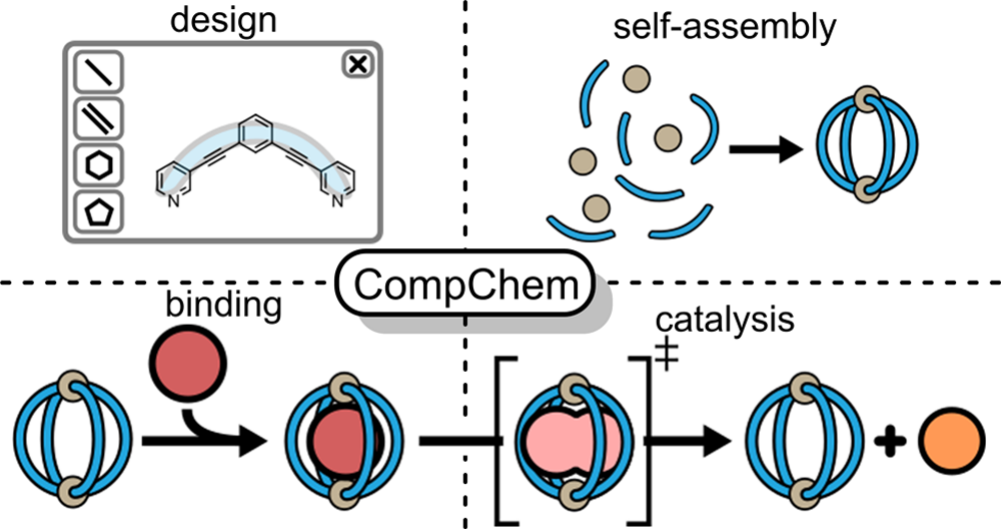

Self-assembled metallo-organic cages have emerged as promising biomimetic platforms that can encapsulate whole substrates akin to an enzyme active site. Extensive experimental work has enabled access to a variety of structures, with a few notable examples showing catalytic behavior. However, computational investigations of metallo-organic cages are scarce, not least due to the challenges associated with their modeling and the lack of accurate and efficient protocols to evaluate these systems. In this review, we discuss key molecular principles governing the design of functional metallo-organic cages, from the assembly of building blocks through binding and catalysis. For each of these processes, computational protocols will be reviewed, considering their inherent strengths and weaknesses. We will demonstrate that while each approach may have its own specific pitfalls, they can be a powerful tool for rationalizing experimental observables and to guide synthetic efforts. To illustrate this point, we present several examples where modeling has helped to elucidate fundamental principles behind molecular recognition and reactivity. We highlight the importance of combining computational and experimental efforts to speed up supramolecular catalyst design while reducing time and resources.

## 1 Introduction

Nature provides stunning examples that show how the organization of relatively simple building blocks leads to vital functions, from compartmentalization to catalysis. Inspired by these observations, chemists have attempted to design artificial structures in the laboratory that can also deliver useful properties. Although these structures are yet to match the performance of nature’s systems, impressive progress has been made in the synthesis of increasingly complex molecular systems, highlighted by the award of the 2016 Nobel Prize in Chemistry for molecular machines.

**Figure 1:**
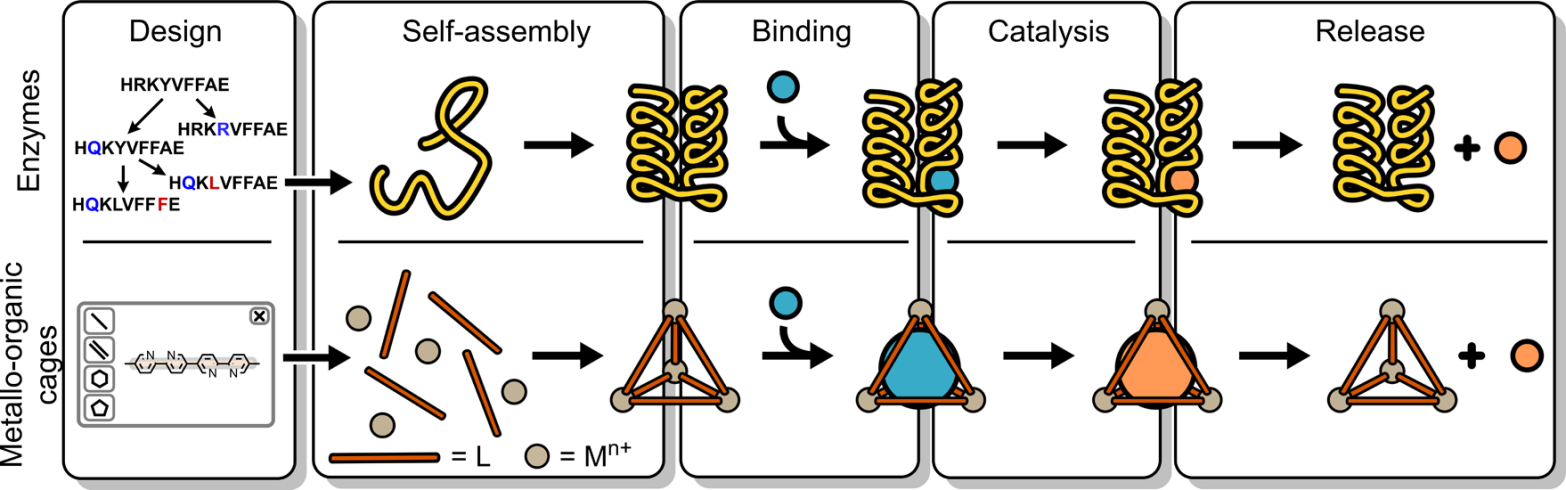
Comparison between enzymes and self-assembled metallo-organic cages. The design of metallo-organic cage catalysts and property prediction requires a detailed understanding of each of the following stages: structural design, self-assembly, binding, catalysis, and release.

Enzymes, in particular, provide inspiration for the design of self-assembled catalysts.^1−3^ Prominent architectures include porous organic cages (POCs)^4,5^ and metallo-organic cages.^6,7^ In particular, metallo-organic cages have emerged as important bioinspired systems due to their tunability and predictable structure, with applications in drug delivery,^8−10^ chemical sensing,^11,12^ recognition,^13−17^ separation,^18−23^ cargo transport,^24^ stabilization of the reactive state,^25−29^ and catalysis.^30^ However, creating metallo-organic cages that mimic the way enzymes work is challenging. This is because enzymes are complex biopolymers containing many different residues that only become functional upon correct folding. The formation of a hollow interior containing a network of noncovalent interactions, such as hydrogen bonds, ion-pairing, and van der Waals interactions, enables enzymes to selectively bind substrates, stabilize transition states (TSs), and achieve catalytic turnover. While metallo-organic cages can mimic several of these features and have the advantage of being easier to (re)design, synthesize, and prepare than enzymes,^31^ it remains challenging to control self-assembly beyond highly symmetric systems (Figure 1).^32,33^ Therefore, current efforts have centered on developing synthetic protocols to obtain cages that are easy to functionalize or with low-symmetry cavities.^34−39^

Alongside new synthetic methods, computational molecular modeling has been employed to investigate structural parameters of metallo-organic cages, such as the volume of the cavity and the metal-to-metal distance. However, more recently, it has also been used to study their binding and catalytic properties, shedding light on the molecular features driving selectivity and activity. Efficient computational tools have also emerged to rapidly predict molecular properties, enabling synthetic chemists to quickly screen multiple cage designs before attempting their synthesis in the laboratory.^40,41^ Combining computational and experimental efforts could substantially speed up the design of functional metallo-organic cages, reducing time and resources.

## 2 From Building Blocks to Catalytic Metallo-organic Cages

As it is the case for enzymes, where folding mainly depends on the sequence of amino acids and their environment, self-assembly is determined by the nature of the ligand(s) and metal(s) building blocks and the experimental conditions. Understanding how assembly takes place becomes particularly challenging when the number of assembling components increases, as many intermediates may be possible. Following assembly, the precise recognition and uptake of a given substrate(s) are determined by the size, shape, and electrostatic complementarity of the host–guest(s) complex. While achieving size and shape complementarity is relatively straightforward employing 3D models, predicting binding and catalysis is much more difficult. This is due to the complexity of the processes involved, which are difficult to characterize experimentally. This has meant that most of the catalysts reported to date have been obtained via a trial-and-error approach and/or chemical intuition.^42−45^ Therefore, for computational chemistry to contribute to the discovery of novel catalysts, it is necessary to develop better computational protocols that can accurately and efficiently quantify solvation, dynamics, and electrostatic effects at the reactant, TS, and product stage. This will, in turn, facilitate experimental design and speed up the identification of new catalysts (Figure 1).

### 2.1 General Self-Assembly Principles

Understanding the design principles underlying metal-driven self-assembly is essential for creating discrete assemblies with well-defined internal environments. Several design strategies have been developed, enabling access to structures with an ever-increasing number of components, albeit often focused on symmetric homoleptic systems that only use two different components, i.e., one ligand and one metal building block.^38^ More recently, strategies to obtain low-symmetry structures, i.e., heteroleptic cages with different organic building blocks, have been developed.

#### 2.1.1 Symmetric Cages

Over the years, a series of synthetic strategies that exploit metal centers as structural building blocks have been introduced to rationalize and design increasingly large and diverse homoleptic structures. Two main approaches include *directional bonding* and *symmetry interaction*, which are based on either control of bonding vectors of the metal precursor or control of the overall symmetry of the components.^46^ While they have illustrated the power of geometrical considerations when designing novel assemblies, a clear-cut division remains challenging, especially when the classification is done a posteriori to rationalize rather than design a given system. Below we briefly describe these strategies and refer the reader to relevant reviews on the topic for further details.^6,7,47^

**Figure 2:**
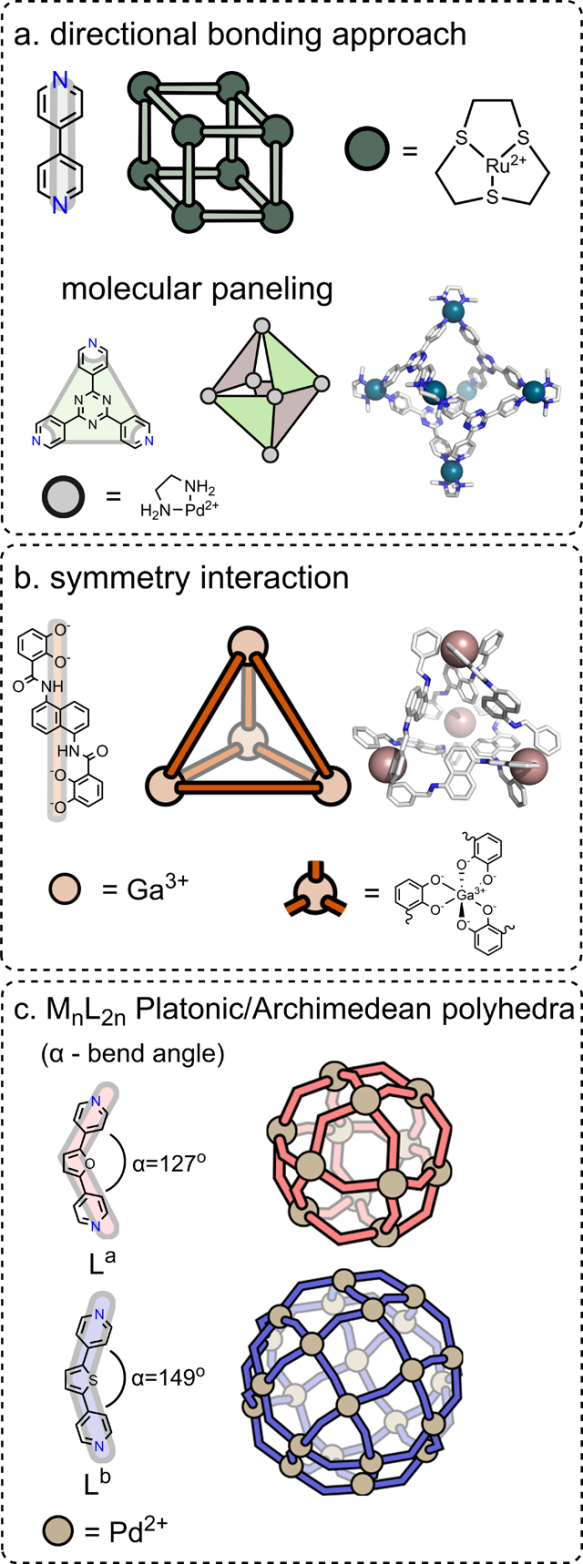
Design strategies for homoleptic cages: (a) directed bonding approach^6,52^ and its variation molecular paneling,^50^ (b) symmetry interaction strategy,^51^ and (c) family of roughly spherical coordination Platonic or Archimedean polyhedra.

The *directional bonding approach* coined by Stang et al.^6^ exploits the use of metallo building blocks to “direct” ligands onto either the edge and/or the face of a polygon or polyhedron (Figure 2a). The outcome of the assembly reaction is mainly determined by the number and relative orientation of the acceptor and donor sites on the metal and ligand, respectively.^6,48^*cis*-Protected square planar complexes are the most widely used metallo component within this method, as the “vacant” coordination sites provide a 90° turn that promotes closure to give a discrete assembly. The strategy was first exemplified by Fujita in his seminal 1990 paper,^49^ which showed that the combination of (en)Pd(NO_3_)_2_ (en = ethylenediamine) and 4,4′-bypyridine leads to a Pd_4_**L**_4_ molecular square in quantitative yield. *Molecular paneling*(50) can be seen as a subset of the directional bonding approach. This method, which employs planar ligands that occupy the faces rather than the edges of the cage, has yielded a number of notable cage structures that possess interesting host–guest and catalytic properties (Figure 2a).

Raymond pioneered a method he defined as the *symmetry interaction* approach to rationalize and predict the outcome of coordination assembly reactions using multibranched catecholate ligands and trivalent pseudo-octahedral metal ions (e.g., Ga^3+^/Fe^3+^).^51^ This method uses ligand design to control the relative orientation of coordination sphere symmetries. For example, a tris(catecholate) metal(III) coordination vertex possesses a *C*_3_-axis that lies perpendicular to the chelate plane. In a M_2_**L**_3_ helicate, the two chelate planes must be aligned in a parallel arrangement, whereas for a M_4_**L**_6_ cage, the ideal angle between any two coordination planes is 70.6°.

This guide is relevant to all cages that are composed of multibranched bidentate ligands and “naked” octahedral metal ions. However, there are multiple instances in which the outcome is different from what would be expected. Assemblies that use tris(bidentate) metal units are interesting (especially from a catalytic perspective) because this coordination sphere is intrinsically chiral (Figure 2b). Often, multimetallic structures are produced as a single diastereomer because strong mechanical coupling influences the Δ or Λ-stereoconfiguration at an adjacent coordination site. For instance, M_4_**L**_6_ cages most commonly, although not always, forms as a 1:1 mixture of ΔΔΔΔ- and ΛΛΛΛ-enantiomers.

Coordination cages that utilize the assembly of “naked” square planar metal ions with ditopic ligands have been classified as the directional bonding approach^7^ but can also be considered using a symmetry interaction description. Indeed, the simplest of these structures, the M_2_**L**_4_ “lantern” cage, can be defined as two MN_4_ coordination planes (where, e.g., N = pyridyl) that are aligned in a parallel arrangement (c.f., M_2_**L**_3_ helicate structure). As the angle between these two planes changes, which is controlled by the bend angle in the bridging ligand, then the size of the [M_*n*_**L**_2*n*_] (e.g., *n* > 2) architecture changes.

Both approaches are underpinned by the same thermodynamic principles, which consider maximum site occupancy (i.e., all metal–ligand interactions are satisfied) and the formation of the smallest, minimally strained structure, maximizing the number of system particles.^53^ With a few exceptions, e.g., square-triangle equilibria or prismatic structures,^6^ the system’s energy minimum is the thermodynamic product. However, for larger cages, kinetic traps can preclude the formation of the predicted lowest energy cage product.^54^

Fujita and co-workers have employed geometry-based design principles to design a series of M_*n*_**L**_2*n*_ Platonic or Archimedean polyhedra (*n* = 6, 12, 24, 30, or 60, Figure 2c).^38^ In this case, unprotected Pd centers were used as precursors in combination with ditopic ligands that, depending on their bend angles, give rise to different size polyhedra. While for the smallest systems (*n* = 6 and 12) the outcome of the assembly reaction follows the predicted product, for larger assemblies kinetic effects play a role.^33^ For example, ligand **L**^**b**^ (α = 149°; Figure 2a), which has a nearly ideal bending angle (α = 150°) to form [Pd_30_**L**^**b**^_60_]^60+^, led to the kinetically trapped [Pd_24_**L**^**b**^_48_]^48+^ cage, which only upon heating was partially converted into [Pd_30_**L**^**b**^_60_]^60+^.^54^ Using a longer ligand, which results in slightly higher flexibility, they later obtained the expected [Pd_30_**L**_60_]^60+^ cage quantitatively. Efforts toward the [Pd_60_**L**_120_]^120+^ cage have also serendipitously led to the self-assembly of a new series of Goldberg polyhedra, including a new topology of [Pd_30_**L**_60_]^60+^ and the giant [Pd_48_**L**_96_]^96+^ cage.^55^ These examples demonstrate that, in addition to geometrical considerations, aspects such as ligand flexibility and experimental conditions also affect the final assembly as they may favor the formation of kinetic traps. Therefore, accounting for these effects in modeling is essential for the successful computational design of cages.

#### 2.1.2 Low-Symmetry Cages

While the strategies mentioned above have led to impressive structures, they are restricted to symmetric cages, often involving just a single ligand, limiting complexity inside the cavity. Synthetic strategies to generate low-symmetry cages have been achieved using either heteroleptic designs based on (i) steric hindrance, (ii) coordination sphere engineering, and (iii) shape complementarity or homoleptic designs (iv) using low-symmetry ligands.^34,35^ Most recent strategies to obtain low-symmetry structures have focused on the [Pd_2_**L**_4_]^4**+**^ “lantern” topology, which is also outlined below.

##### Steric Hindrance

**Figure 3:**
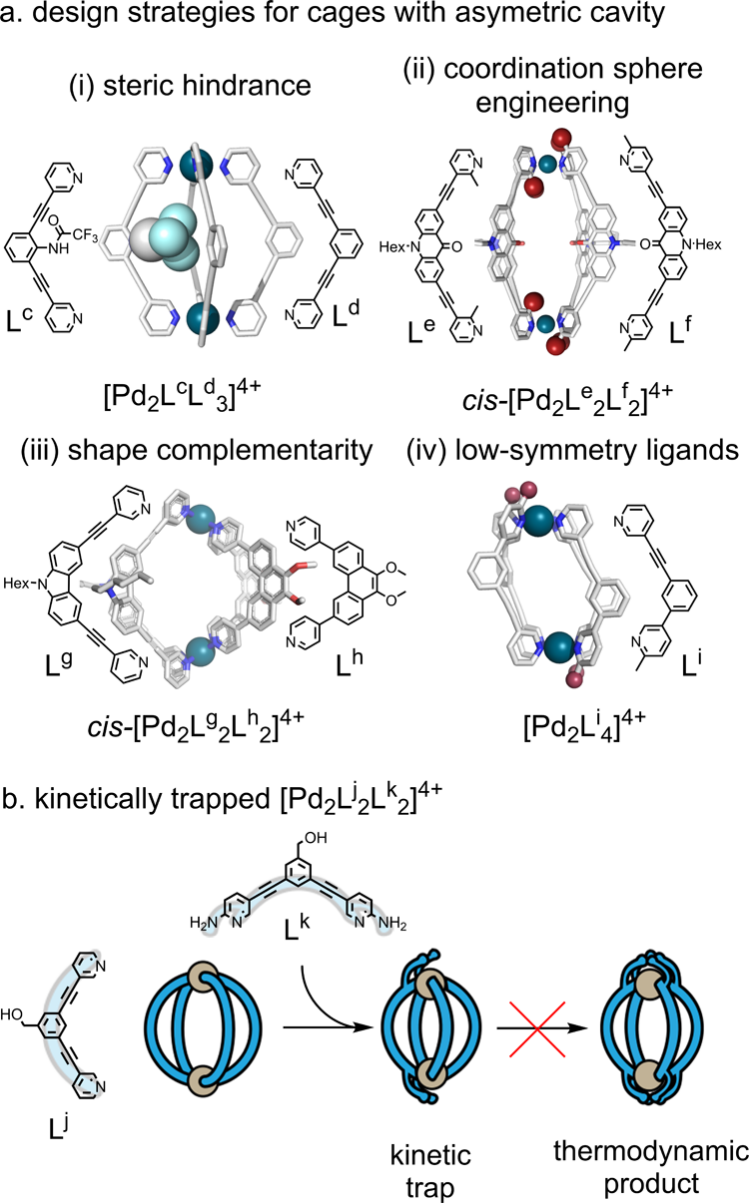
Design strategies to obtain low-symmetry cages. (a) Thermodynamic control: (i) steric hindrance, (ii) coordination sphere engineering, (iii) shape complementarity, and (iv) homoleptic cages via low-symmetry ligands. Red spheres indicate the methyl group proximal to the metal ion. (b) Kinetic control.

Hooley and Johnson have exploited steric hindrance between endohedrally modified ligands to obtain heteroleptic cages. Using a mixture of bispyridyl ligands, **L**^**c**^ and **L**^**d**^, they obtained the heteroleptic [Pd_2_**L**^**c**^**L**^**d**^_3_]^4**+**^ cage (Figure 3a(i)).^56^ One of the main disadvantages of this method is that the functional group occupies the cavity of the cage, which blocks the binding of guests. Clever and co-workers have shown that appending ligands with steric bulk does not necessarily lead to cages with blocked cavities. They showed that an unusual [Pd_4_**L**_4_**L**′_4_]^8+^ tetrahedral cage could be formed from a mixture of exohedrally modified and nonmodified ligands by balancing the entropic tendency to form smaller assemblies and repulsion between bulky functional groups, resulting in low-symmetry unoccupied cavities.^57^

##### Coordination Sphere Engineering

The coordination sphere engineering approach uses substituted ligands that, due to steric or noncovalent interactions around the coordinating atom, disfavor the formation of homoleptic cages. Fujita and co-workers pioneered this strategy and demonstrated that prismatic assemblies that incorporate ditopic and tritopic ligands could be favored by exploiting a sterically hindered 2,6-dimethyl-subsituted pyridyl motif, which stopped self-sorting.^58^ Clever and co-workers have also employed this strategy to assemble a heteroleptic *cis*-[Pd_2_**L**^**e**^_2_**L**^**f**^_2_]^4**+**^ cage from 6- or 2-methyl-substituted ligands.^59^ When the homoleptic [Pd_2_**L**_2_]^4+^ assembly with ligands **L**^**e**^ or **L**^**f**^ was attempted, steric hindrance around the metal center disfavored cage formation; instead, [Pd_2_**L**_3_(solvent)_2_]^4+^ and [Pd_2_**L**_2_(solvent)_4_]^4+^ structures were generated under kinetic control. When the combination of ligands **L**^**e**^ and **L**^**f**^ was used, the “in/out” orientation of methyl groups resulted in the formation of *cis*-[Pd_2_**L**^**e**^_2_**L**^**f**^_2_]^**4+**^ cages under thermodynamic control (Figure 3a(ii)).

##### Shape Complementarity

In this strategy, two shape-complementary ligands are combined, resulting in enthalpic destabilization of the homoleptic species and, in some cases, entropy reduction of the heteroleptic cage due to size reduction. Zhou and Li originally employed this approach in a series of Cu^II^-based cages,^60^ where the heteroleptic cage was achieved via ligand displacement from a homoleptic cage. Clever and co-workers have also employed this approach to obtain thermodynamically stable *cis*-[Pd_2_**L**^**g**^_2_**L**^**h**^_2_]^4+^ and *trans*-[Pd_2_**L**^**g**^_2_**L**_2_]^4+^ heteroleptic cages either from the mixture of precursors or via ligand substitution from their homoleptic cage precursors (Figure 3a(iii)).^61^

##### Low-Symmetry Ligands

Low-symmetry homoleptic cavities can also be obtained using low-symmetry ligands.^62−65^ This strategy uses coordination sphere engineering and/or shape complementarity introduced in a single ligand. For example, Lewis and co-workers employed a low-symmetry ligand containing a 2-substituted pyridyl donor to generate low-symmetry [Pd_2_**L**^**i**^_4_]^4+^ cages for which four different isomeric forms exist. The increased steric hindrance around the metal center and the use of different linker lengths resulted in misalignment around the metals, favoring the formation of the unsymmetrical cage (Figure 3a(iv)).^36^

While most unsymmetric cages are formed using one of the strategies described above, other notable examples exist. For example, Crowley and co-workers have studied the sequential substitution of ligands by reacting the [Pd_2_**L**^**j**^_4_]^4+^ cage with 2-amine-substituted bispyridyl ligand, **L**^**k**^.^66^ Rather than observing the expected [Pd_2_**L**^**k**^_4_]^4+^ thermodynamic product, they obtained the kinetically trapped heteroleptic [Pd_2_**L**^**j**^_2_**L**^**k**^_2_]^4+^ cage (Figure 3b), which did not undergo further exchange after being left for 40 days at room temperature. Presumably, the amino groups of the ligands shield the palladium ion and prevent ligand exchange. Obtaining kinetically trapped cages could be an alternative strategy for designing heteroleptic cages, but it remains an unexplored direction in the field.^67^

### 2.2 Computational Cage Design

In recent years, the computational prediction of synthetically viable structures via in silico screening has become increasingly popular,^68−71^ complementing the synthetic strategies described above. This has been possible thanks to the growth in computational power and algorithmic improvement of modeling software. Different modeling techniques are currently available, where the choice of the method depends on the size of the system and process under study and the resources available. These approaches can be generally divided into classical molecular mechanics (MM) and quantum mechanics (QM) approaches.

#### 2.2.1 Classical Approaches

Classical force fields (FFs) describe atoms as charged points with Lennard-Jones interactions linked by springs representing bonds, allowing the evaluation of potential energies with a simple and computationally efficient algorithm. As a result, systems with millions of atoms can be simulated on millisecond time scales.^72^ Several force fields exist for describing organic molecules, including the universal force field (UFF),^73^ the general AMBER force field (GAFF),^74^ the CHARMM general force field (CGenFF),^75^ the optimized potentials for liquid simulation force field (OPLS),^76^ and the OpenFF family.^77^ The UFF includes parameters for most atoms of the periodic table (including metals) and has also been extended to metal–organic frameworks (MOFs, UFF4MOF).^78^ These FFs have been carefully parametrized to reproduce, for example, hydration free energies, partition coefficients, QM energy profiles, or vibrational frequencies, often targeting biological systems; however, their intrinsic simplicity means that they provide limited quantitative estimates of, for example, binding free energies.^79^ Moreover, while classical FFs can describe noncovalent interactions and self-assembly, they do not allow the study of the formation or breaking of covalent bonds, making them unsuitable for studying chemical reactions and catalysis. Indeed, only a few exceptions exist, such as ReaxFF and the empirical valence bond (EVB) approach, which require extensive parametrization.^80,81^

Modeling metal-containing systems using current FFs is particularly challenging, as FFs often lack parameters for metal centers or even protocols to obtain them. Moreover, when they exist, problems associated with their stability during molecular dynamics (MD) simulations often appear. For example, metals may strongly interact with counterions or repel other metals centers nearby.^82^ Three main protocols have been reported to model metal ions classically; most of them aim to reproduce aquo complexes geometries and solvation free energies. They include the commonly used *soft-sphere model*,^83,84^ in which the metal–ligand interactions are described through electrostatic and van der Waals terms only. While these models are simple to parametrize, they are unable to simultaneously reproduce two or more experimental properties, e.g., first solvation shell and hydration free energy.^84^ The *covalent bond model* includes predefined covalent bonds between the metal and ligands, which enhance stability; however, it precludes ligand exchange. The Seminario method,^85^ automated in MCPB.py protocol,^86^ is often used to obtain bonded parameters. To account for charge transfer between the ligand and the metal, partial charges are also recomputed. Finally, the *dummy model* describes the metal center as a set of cationic dummy atoms placed around the metal nucleus, encouraging a specific coordination geometry on the metal center.^87^ Since this model allows for breaking metal–ligand bonds, it is the model of choice for self-assembly studies.^88−90^ As described below, all these methods have been used to model metallo-organic cages with different levels of success.

#### 2.2.2 Quantum Approaches

To reliably quantify the origin of catalysis, ab initio (wave function-based) or density functional theory (DFT) methods are necessary. They allow the optimization of geometries and the calculation of energies and relative (free) energies. However, their applicability is limited; even low-cost DFT methods (e.g., B97-3c^91^ and PBEh-3c^92^), which can be applied for energy calculations with up to 1000 atoms, are computationally impractical for larger systems.^92^ Semiempirical QM methods, such as PM*x* methods^93−95^ and the more recently developed extended tight-binding methods of the xTB family,^96,97^ provide an efficient alternative to optimize large structures. For instance, the xTB family enables optimization and thermochemistry evaluation of systems with up to 1000 atoms, including metal centers. In the field of metallo-organic cage modeling, currently used methods include DFT calculations to quantify the relative stability of cage conformers/isomers with structures optimized at either the DFT,^66,98^ PM*x*,^27,63,99−101^ or xTB^36,102^ level of theory.

#### 2.2.3 Automated Tools

In the last 10 years, there has been enormous progress in open-source software development, including Open Babel^103^ and RDKit,^104^ which facilitate 3D conformer generation and determination of ground-state properties, such as geometries, charges, and dipoles. Indeed, several open-source tools are now available for high-throughput screening of COFs, MOFs, rotaxanes, and metallo-organic cages.^40,41,105−107^ They commonly employ classical force fields or semiempirical-based algorithms for fast generation of structures allowing the creation of an extensive library of scaffolds, which subsequently is reduced by a series of filters to structures with desired properties.

**Figure 4:**
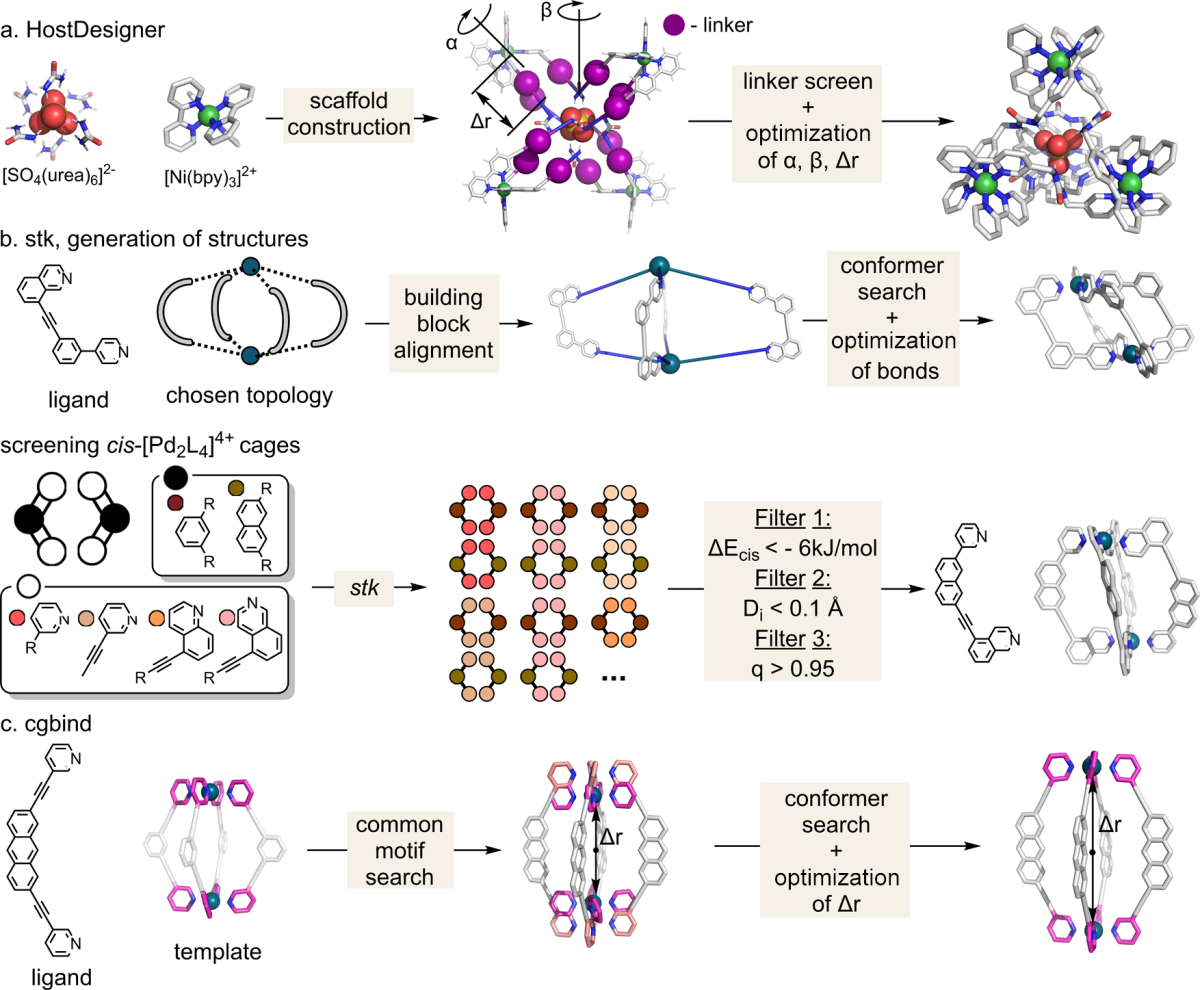
High-throughput screening. (a) *HostDesinger* procedure to generate a cage with high affinity toward SO_4_^2–^, (b) *stk* procedure for cage generation and its use in the identification of low-symmetry *cis*-[Pd_2_**L**_4_]^4+^ cages, and (c) *cgbind* procedure for cage generation.

The computational tool *HostDesigner* has been developed by Hay and Firman^108^ to design new hosts that can effectively bind cations^109^ and anions.^110^ The authors designed a sulfate host by mimicking the interaction that the anions establish with water (Figure 4a).^111^ Their calculations indicated that sulfate forms up to 12 hydrogen bonds with water; these interactions were mimicked with six urea molecules that formed a T-symmetry [SO_4_(urea)_6_]^2–^ complex. They then employed [Ni_4_**L**_6_]^8+^ whereby [Ni(*bpy*)_3_]^2+^ molecules occupy the vertices of the tetrahedron with sulfate in the center. By simultaneously screening linkers and varying the positions and orientations of the vertices and complex, they designed and synthesized a cage with a higher affinity toward sulfate than any available sulfate receptor synthesized to date.

Two prominent open-source cage generation tools with graphical interfaces include *stk* developed by Jelfs and co-workers^40^ and *cgbind* developed by our group.^41^ Both tools generate cages by providing ligands with specified and predefined topology. In their current form, they do not allow predicting the lowest energy architecture for specified ligand(s). *stk* was originally designed to generate structures of small linear polymers, porous organic cages, and covalent organic frameworks (Figure 4b).^40,112^ However, it has also been extended to rotaxanes, host–guest complexes, metallo-organic cages, and MOFs.^40^ The tool uses predefined topological graphs, where the building blocks are placed on the edges and the vertices of the graph. They are then joined by bonds, and in the case of covalent organic molecules, redundant atoms are removed. To ensure that atoms do not overlap, the assembly initially has bonds with exaggerated distances, which are then energy minimized using third-party optimizers, such as RDKit, xTB, Schrödinger’s Macromodel, or GULP,^113^ or their Monte Carlo based MCHammer optimization.^114^

Lewis, Jelfs, and co-workers have used *stk* in combination with UFF4MOF and xTB to screen low-symmetry *cis*-[Pd_2_**L**_4_]^4+^ cages.^102^ A library of 60 ligands, which generated 240 cages, was screened using three filters. First, isomers with energies >6 kJ mol^–1^ relative to the lowest energy isomer were disregarded. To ensure that dinuclear structures containing a square planar metal configuration were preferred over multinuclear ones, two criteria were measured: the sum of the distance of four nitrogen atoms from the plane defined by the PdN_4_ unit and a square planar order parameter.^115^ As a result, five out of 60 ligands were synthesized, and four of them successfully formed a clean *cis*-isomer, which was confirmed by NMR and DOSY experiments.

Our computational tool, *cgbind*,^41^ targets the generation of metallo-organic cages from crystal-structure templates. The approach is based on finding a common motif of donor atoms in a template and input ligand. The optimal structure is found by screening different conformations of ligands and optimizing the distance of the position of the metal from the center of the cage (Figure 4c). Several frameworks are implemented in the code (including M_2_**L**_4_ and M_4_**L**_6_), while new ones can be added by providing a structure downloaded from the CSD or generated by a molecule editor, e.g., Spartan^116^ or Avogadro.^117^ The cages generated by this method have shown an excellent agreement with crystal structures (root-mean-square deviation (RMSD) < 1.5 Å). Additionally, the structures can be further optimized by xTB, MOPAC, ORCA, or NWChem by interfacing *cgbind* to *autodE*.^118^*cgbind* also includes a series of analysis tools, such as a maximum enclosed and escape sphere and electrostatic potential surface. Moreover, it provides a fast and straightforward way to optimize the position of substrates inside the cavity and estimate the binding affinity using a simple encoded nonbonded force field. In addition to the Python module, a limited version of *cgbind* is available as a web-based graphical user interface at cgbind.chem.ox.ac.uk.

*HostDesinger*, *stk*, and *cgbind* rely on covalently connected ligands and metals of predefined architectures to reduce the configurational space to search. As a result, some of the generated cages might have unreasonable structures. Although they can be improved, for example, by geometry optimization with xTB, this would significantly increase computational cost when exploring increasingly large systems. Moreover, the methods do not consider interactions with solvent or ions, flexibility of ligands, and entropic contributions. Therefore, they only provide information about the final structure and not their likelihood (kinetic or thermodynamic driving force) to form under specific experimental conditions. These factors could be considered, for example, by using MD simulations. However, simulating self-assembly from metal and ligand precursors might require microsecond-long MD simulation (Section 3.2),^89,119^ making the approach impractical for routine cage design.

Another consideration is accessibility of the software to a broader scientific audience with less computational experience. Most of the described software is command-line and therefore requires basic programming skills (with the exception of the web-based version of *cgbind*). Development of the graphical user interfaces is needed for broader applicability of these methods.

#### 2.2.4 Architecture Prediction

Reek and co-workers have employed classical modeling to predict the preferential cage architecture of homo- and heteroleptic cages with four different ligands.^120^ They employed GAFF to describe the organic ligands and a covalent bond model to describe the metal center, with Lennard-Jones parameters obtained from a soft-sphere model.^84^ Bonded parameters between the metal and nitrogen donor were fitted to reproduce DFT energies for configurations generated from xTB. To account for charge transfer between the donor nitrogen and the metal, the RESP method was employed,^121^ which assigned a +0.26 *e* charge to Pd. Acetonitrile solvent was modeled implicitly using the Generalized Born model.^122^ The ligands were mapped into predefined templates, representing existing and hypothetical polyhedral M_*n*_**L**_2*n*_ architectures with *n* = 3–30 vertices. The relative distribution of cages was then evaluated by Boltzmann weighting.

**Figure 5:**
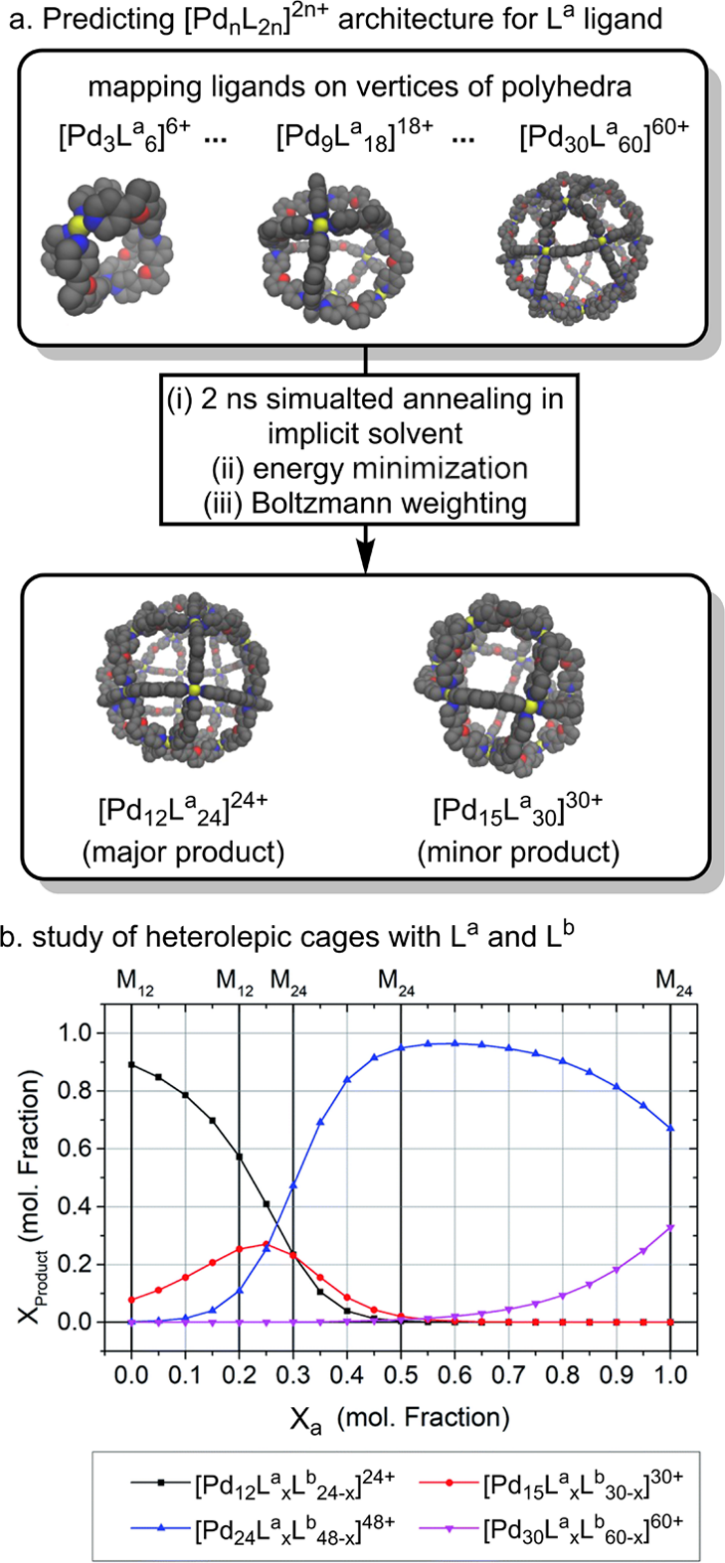
Prediction of the [Pd_*n*_**L**_2*n*_]^2*n*+^cage architectures.^120^ (a) Prediction of homoleptic cages for the **L^a^** ligand. (b) Architecture prediction of heteroleptic [Pd_*n*_**L**^**a**^_*x*_**L**^**b**^_2*n*–*x*_]^2n+^ cages for a mixture of **L^a^** and **L^b^** ligands. Adapted from ref (120) with permission from the Royal Society of Chemistry.

The procedure was first tested for the **L**^**a**^ ligand, originally reported by Fuijta and co-workers to form a single topology, [Pd_1__**2**_**L**^**a**^_24_]^24+^ (Figure 2a).^123,124^ Reek and co-workers reproduced these results computationally, demonstrating the formation of [Pd_12_**L**^**a**^_24_]^24+^ as a major product (89.1%).^120^ However, the model suggests the presence of a minor assembly, [Pd_15_**L**^**a**^_30_]^30+^ (Figure 5a), which could be experimentally detected via mass spectroscopy.

The same approach was then extended to study assemblies for various molar fractions of **L**^**a**^ and **L**^**b**^ ligands, which are known to form [Pd_12_**L**^**a**^_*x*_**L**^**b**^_24–*x*_]^24+^ heteroleptic cages.^123^ The authors suggested a thermodynamic preference for [Pd_12_**L**^**a**^_*x*_**L**^**b**^_24–*x*_]^24+^ cages when up to 0.27 mole fraction of **L**^**a**^ was used, while for a higher molar fraction of **L**^**a**^, the [Pd_24_**L**^**a**^_*x*_**L**^**b**^_48–*x*_]^48+^ cage was preferred. This is in line with previous experimental reports (Figure 5b).^123^

This protocol also successfully predicted the formation of homoleptic cages formed from endo- and exohedrally modified ligands; however, it failed to predict the correct architectures when using different ratios of ligand mixtures, for which larger assemblies are expected. The authors associated this failure with the differences in the ligand’s dihedral angles, which were only captured by DFT but not by the classical model.

## 3 Self-Assembly Mechanism

While design rules enable one to assess the thermodynamic stability of the desired assembly, they do not guarantee that such a structure can be isolated or even observed, as kinetic traps may prevent the thermodynamic product from being reached. A mechanistic understanding of self-assembly could help the rational design of novel cages by identifying competing pathways, potential kinetic traps, and interconversion barriers. However, obtaining information using experimental techniques is challenging as it is often extremely difficult to detect (and reliably characterize) early-stage intermediates using noninvasive, quantifiable methods. For example, metal–ligand assemblies that form at the beginning of a reaction are not only present in low concentration, but they are also often low-symmetry structures, which makes their NMR signal weak. This contrasts with the NMR signals observed for the final closed structure, where a single resonance corresponds to many atoms in equivalent chemical environments. Therefore, computational modeling provides a promising avenue to fill this gap, complementary to experiments.

### 3.1 Experimental Approaches to Quantify the Self-Assembly Reaction Pathway

**Figure 6:**
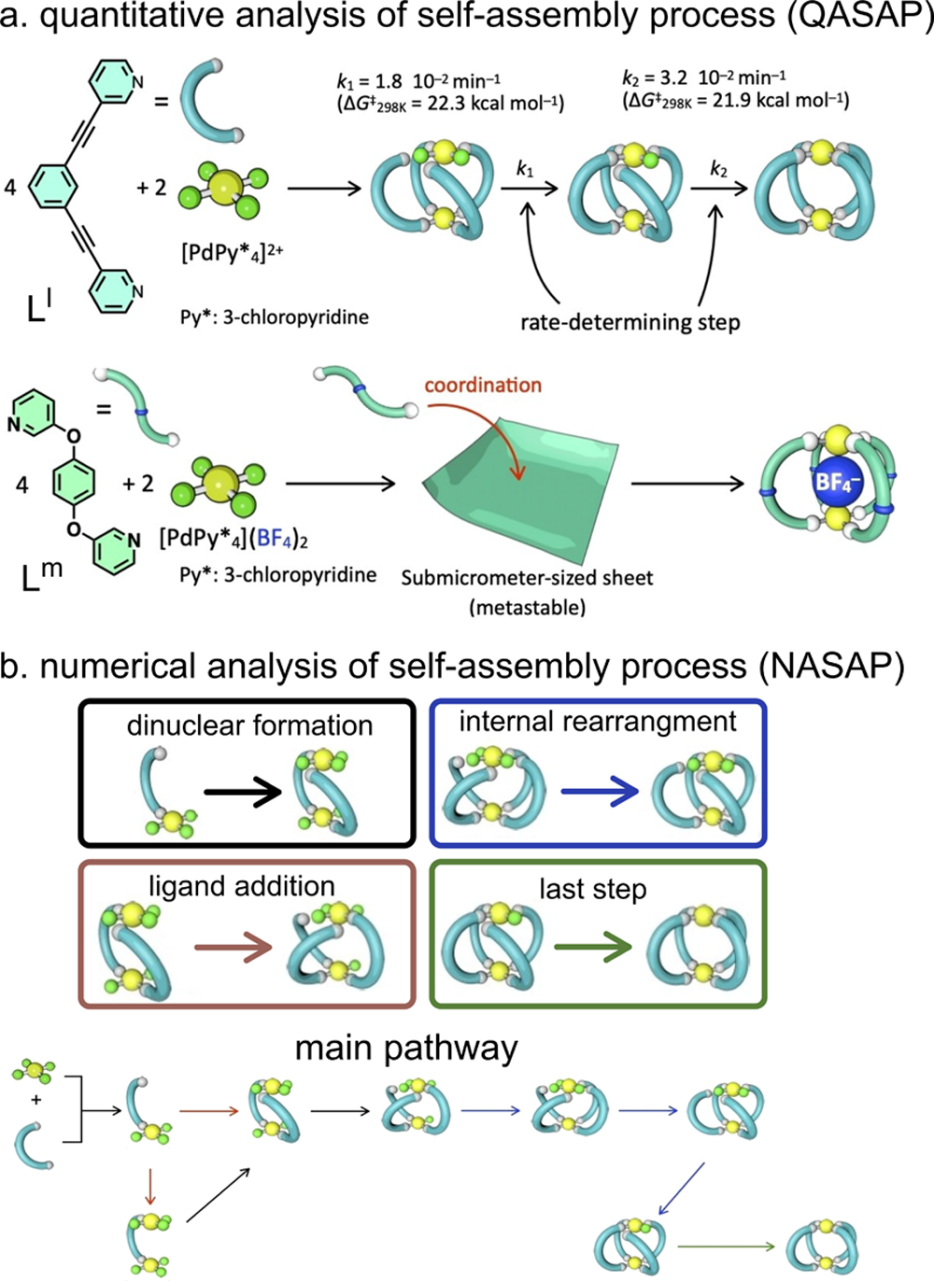
QASAP and NASAP approach. (a) Assembly mechanism deduced using the QASAP approach for [Pd_2_**L^l^**_4_]^4+^ and [Pd_2_**L^m^**_4_]^4+^ cages.^128,131^ (b) Assembly mechanism of the [Pd_2_**L**^**l**^_4_]^4+^ cage elucidated using the NASAP approach. The assembly was classified into four stages to infer the main assembly pathway. Adapted from ref (126) with permission from the Chemical Society of Japan and Wiley-VCH GmbH, Weinheim.

There are only a few experimental reports exploring the assembly mechanism of metallo-organic cages. Most notably, Hiraoka and co-workers have developed the quantitative analysis of the self-assembly process (QASAP) approach.^125,126^ This method indirectly provides information about intermediate structures from measurable data, most commonly the amount of a precursor ligand that is displaced during the course of the reaction.^127−131^ QASAP relies on calculating two parameters from the species that can be measured by NMR: *n*—the average number of metal ions bound to ligand and *k*—the metal/ligand ratio. From average (*n, k*) values, the progression of the sample’s composition and the presence of intermediates can be inferred. This technique has been used to study the self-assembly of [Pd_2_**L**_4_]^4+^,^127−131^ [Pd_3_**L**_6_]^6+^,^132^ [Pd_4_**L**_8_]^8+^,^133,134^ [Pd_6_**L**_8_]^12+^,^130^ [Pd_12_**L**_24_]^24+^,^135^ [Pt_6_**L**_6_]^12+^,^136^ and [Pt_3_**L**_3_]^6+^/[Pt_6_**L**_6_]^12+^.^137^ For instance, the formation of a [Pd_2_**L**^**l**^_4_]^4+^ cage from [Pd(Py*)_4_]^2+^ (Py* = 3-chloropyridine) and rigid ditopic ligands was found to proceed via intermediates [Pd_2_**L**^**l**^_4_(Py*)_2_]^4+^ and [Pd_2_**L**^**l**^_4_Py*]^4+^.^131^ By monitoring (*n*, *k*) values, it was estimated that all the building blocks were consumed within the first 5 min, after which a steady increase of final product was observed via the identified intermediates (15–300 min) (Figure 6a). Mass spectroscopy experiments also confirmed the presence of these intermediates. The authors also obtained the activation energy barriers for the formation of the first (Δ*G*_1_^‡^ = 22.3 kcal mol^–1^) and second (Δ*G*_2_^‡^ = 21.9 kcal mol^–1^) intermediate, in reasonable agreement with DFT calculations (Δ*G*_1_^‡^ = 17.5 kcal mol^–1^ and Δ*G*_2_^‡^ = 17.7 kcal mol^–1^, respectively). When the same approach was applied to studying the formation of the [Pd_2_**L**^**m**^_4_]^4+^ cage from a flexible ligand, **L**^**m**^ (Figure 6a),^128^ it was found that the product was slowly formed via a submicrometer-sized sheet intermediate rather than the intermediates described above. This state was characterized by dynamic light scattering (DLS) and transmission electron microscopy (TEM).^128^

Sato and Hiraoka have also introduced a numerical analysis of the self-assembly process (NASAP),^126,138^ which complements the QASAP approach by providing information on intermediates at an early stage of self-assembly (<5 min, Figure 6b). NASAP identifies the major assembly pathways using a graph representation and the Gillespie stochastic simulations,^139^ and it has been used to probe the formation of [Pd_2_**L**_4_]^4+^,^67,140,141^ [Pd_3_**L**_6_]^6+^,^142^ and [Pd_6_**L**_4_]^12+^ cages.^138,143,144^ For [Pd_2_**L**^**l**^_4_]^4+^, 29 intermediate species, 68 elemental reactions, and four pathways were analyzed.^140^ Only pathways with small-sized intermediates (M_*n*_**L**_*m*_, *n* ≤ 2 and *m* ≤ 4) were considered. For this system, the analysis showed that assembly starts with the formation of [Pd_2_**L**^**l**^_2_(Py*)_5_]^4+^, which then leads to [Pd_2_**L**^**l**^_4_(Py*)_3_]^4+^ and finally rearranges to the final product.

Both QASAP and NASAP have enabled a better understanding of the self-assembly mechanism and, consequently, the rational access to kinetically trapped cages. For example, [Pd_2_**L**_4_]^4+^ cage^67^ and [Pd_6_**L**_4_]^12+^ square-based pyramid^144^ structures have been obtained using this information rather than serendipity. Both methodologies also demonstrated the importance of counteranions in the self-assembly pathway. For example, the expected cage was only formed when the templating guest BF_4_^–^ was present; in contrast, only uncharacterized byproducts were obtained in the absence of any templating anion.^67^

### 3.2 Computational Approaches to Explore Self-Assembly

Yoneya and co-workers pioneered the computational modeling of the metallo-organic cage assembly. They have used stochastic MD simulations and implicit solvation to study the formation of [Pd_6_**L**_8_]^12+^ and [Pd_12_**L**_24_]^24+^ cages.^89,119^ To describe the ligands, they used a united-atom model, where hydrogen atoms are merged into bonded non-hydrogen atoms, and a dummy model for Pd^2+^, which was parametrized to reproduce the crystallographic Pd–N distance. This simplified model substantially reduced the number of degrees of freedom and enabled them to reach a microsecond time scale. Long-range electrostatic interactions were computed by the generalized reaction-field method with a relative dielectric constant of the solvent (ε_r_ = 47; DMSO).^145^ To model the [Pd_6_**L**_8_]^12+^ cage, short-range electrostatic interactions were screened with various relative dielectric constants (ε_r_ = 1.0, 2.5, and 4.0) to determine the optimal one for the process under study. When a dielectric constant of ε_r_ = 1.0 (vacuum) was employed, the ligand–metal interaction was so strong that disassembly was extremely rare, preventing the correction of initially formed kinetic traps. On the other hand, employing a dielectric constant of ε = 4.0 led to a fast ligand–metal exchange that made it impossible to form any ligand–metal complex. Only for ε = 2.5, the exchange rate of ligand and metal provides a balance between growth and disassembly, allowing cage formation and correction of structural defects. Subsequently, explicit DMSO molecules were also added to the system. This resulted in a shortening of the relative lifetime of small assemblies compared with the complete nanosphere. The simulations did not include counterions as they affected the stability of the metal model employed.

In subsequent work, the same authors studied the formation of two larger homoleptic [Pd_12_**L**_24_]^24+^ cages from ligands with different bend angles.^124^ In comparison to their previous study, a higher number of kinetic traps—closed structures with lower nuclearity than [Pd_12_**L**_24_]^24+^, i.e., [Pd_6_L_12_]^12+^, [Pd_8_**L**_16_]^16+^, and [Pd_9_**L**_18_]^18+^—were observed. Moreover, furan-cored ligands with slightly larger bend angles led to fewer kinetic traps, as the formation of a small cluster increased strain. Modifying atomic charges to mimic charge transfer also affected the resulting structures, further stabilizing the complexes. These results illustrate the challenges when attempting to evaluate the kinetics of assembly processes, which can be highly dependent on the dielectric of the environment, the presence of explicit solvent molecules, and the consideration of charge transfer. As mentioned above, these aspects are often evaluated via trial and error to balance ligand exchange and assembly events on a reasonable time scale.

Tan and co-workers have also performed simulations in implicit solvent to study the formation of a [Hg_2_**L**_4_]^4+^ cage.^146^ This cage experimentally assembles in acetonitrile and has been found to encapsulate C_60_ and C_70_, with the [C_60_⊂Hg_2_**L**_4_]^4+^ complex being the most stable. The guest can be removed upon the addition of Hg^2+^ ions, leading to the formation of the [Hg_2_**L**_2_]^4+^ metallocycle.^21^ Similar to Yoneya’s approach, Hg^2+^ was described with a dummy model with parameters obtained to reproduce the Hg–N distances and N–Hg–N angle in [Hg_2_**L**_2_]^4+^. Organic molecules were modeled with the GAFF force field, and simulated annealing was employed to speed up conformational sampling and promote guest encapsulation. The authors identified a stepwise assembly from the [Hg_2_**L**_2_]^4+^ metallocycle to the [Hg_2_**L**_3_]^4+^ intermediate and finally to the [Hg_2_**L**_4_]^4+^ cage.

While metallo-organic cage self-assembly remains challenging to model due to the difficulties in reliably modeling metal centers, it is expected that advances in force-field development will enable more realistic simulations with explicit solvent and counterions. For example, it would be interesting to combine MD with rare-event sampling methods, such as metadynamics,^147,148^ or Markov state models,^117^ to quantify the kinetics of the assembly process. These approaches have been used, for example, by Hiraoka and co-workers to study the solvent effect in the self-assembly of an organic cage formed from six aromatic ligands in aqueous methanol^149,150^ but not yet to model metallo-organic cage assembly. In the cited example, Markov state models were used to study the assembly pathway and estimate the rate-limiting step.

Applying similar techniques to study metallo-organic cage formation would provide valuable information about the formation of kinetic traps and their rate of interconversion, which could be exploited for unsymmetric cage design. Moreover, they could complement more demanding experimental approaches, such as QASAP and NASAP. However, for these approaches to become predictive and accurate, the description of solvent and counterions is required, as they will affect the stability of intermediates and their rate of exchange. It is worth noting that the composition of the system depends exponentially on the relative energy difference, and therefore high-accuracy FFs are required to determine the ratio of the products.

## 4 Binding and Guest Release

### 4.1 Experimental Studies

#### 4.1.1 Key Factors Controlling Host–Guest Interactions

The ability of a guest to bind to a given cage depends on a number of parameters, including size, shape, and electrostatic complementarity, as well as other factors such as the nature of counterions and solvation. Metallo-organic cages are also invariably charged, and this can have significant implications.

Rebek introduced the “55% rule” to predict host–guest binding—it states that the optimal volume of the guest should be around 55 ± 9% of the internal host volume.^151^ While originally developed for organic capsules, this rule-of-thumb has also been used with metallo-organic cages.^17,152,153^ However, caution has to be taken when using this rule. This is because the volume of organic capsules is often easier to define. In contrast, metallo-organic cages may possess several windows making the calculation of their internal volume challenging, as it is difficult to define the boundary between the inner cavity and bulk solvent. This possibly explains why the 55% rule has been applied to metallo-organic cages with varying success.^153−155^

Counterions play an enormous role in the host–guest chemistry of metallo-organic cages. Often, one or several counterions act as strong binding guest(s); this is common with small tetrahedral cages that are often shape or size complementary for common weakly coordinating anions (e.g., BF_4_^–^, PF_6_^–^) that are often used in self-assembly reactions.^156,157^ Counterions can also influence the solubility of metallo-organic cages. For example, small, charge dense counterions that can strongly hydrate have been used to create water-soluble systems with both cationic cages (using oxyanions such as nitrate) and anionic cages (e.g., use of K^+^ in [Ga_4_**L**_6_]^12–^). Externally hydrated counteranions allow the cage to bind less polar guests, which can be significantly enhanced by the hydrophobic effect. This approach has been particularly successful with cages that possess flat aromatic panels because these can create a solvophobic cavity. Water-soluble cage systems have been particularly relevant for catalysis as they facilitate the binding of organic substrates.

Engineering the cage system so it remains free for binding organic substrates has also been accomplished using large counterions that cannot access the internal cavity. Our group has adopted this approach, exploiting tetrakis[3,5-bis(trifluoromethyl)phenyl]borate (BArF_4_^–^) counteranions.^158^ This strategy has an opposite effect on solubility; it allows charged, multimetallic cages to be used in apolar solvents such as dichloromethane. This also means that the cavity acts as a polar environment in comparison to the solvent phase (c.f., hydrophobic guest binding with water-soluble cages). As binding is driven by the formation of polar host–guest interactions rather than solvent–solvent interactions, this maximizes the opportunities to leverage catalytic activity using electrostatic effects.

#### 4.1.2 Mechanism of Guest Binding

**Figure 7:**
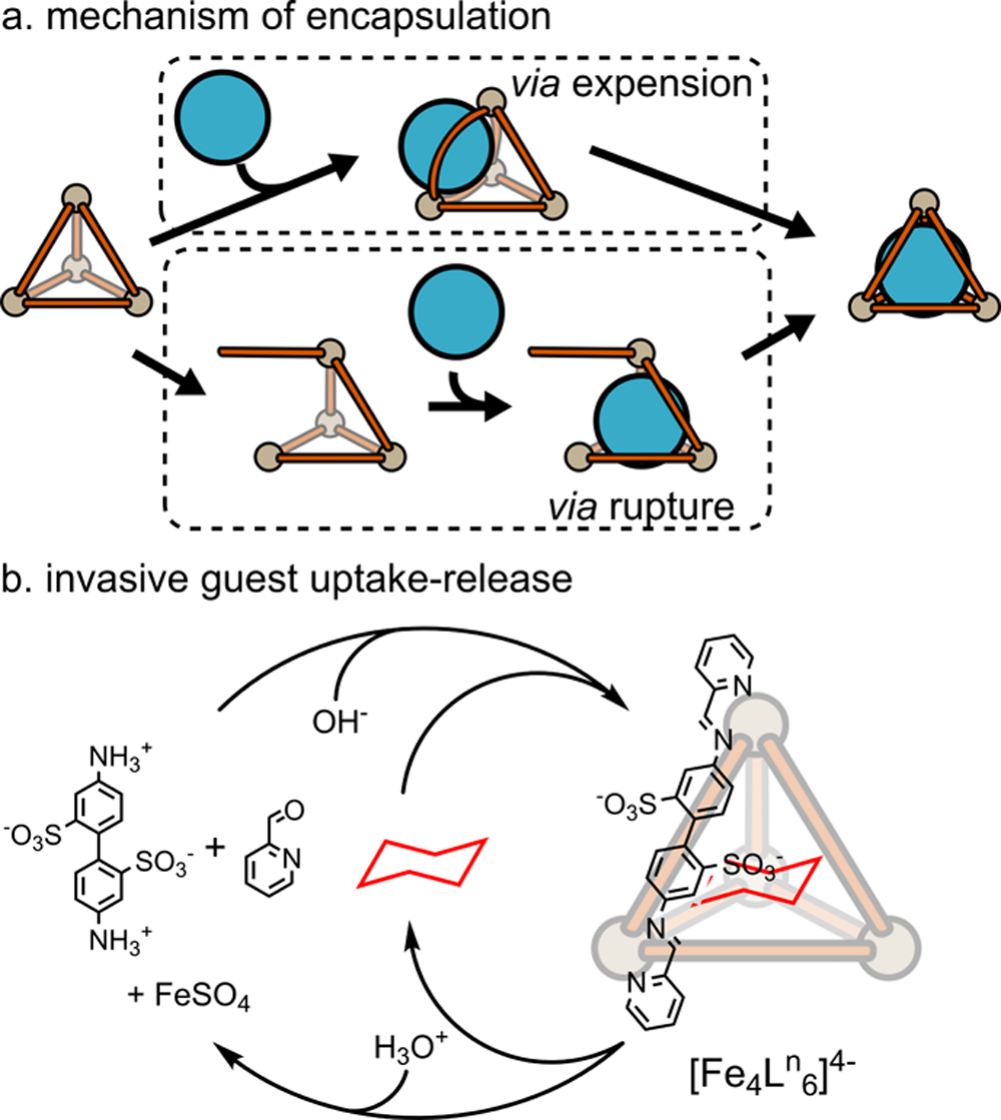
Binding of guests. (a) Uptake of the guest via expansion of the cage or (partial) rupture of the cage. (b) Example of controlled uptake and release of the guest involving the complete disassembly of the cage.

Guest binding and release can be achieved via a series of mechanisms involving the expansion and partial or complete disassembly of the cage. For example, Raymond and co-workers employed bulky guests (NEt_4_^+^ and PPr_4_^+^) within [M_4_**L**_6_]^12–^ cages (M = Ga^3+^, Ti^4+^, Ge^4+^).^159,160^ Those guests were much larger than the size of their window sizes; however, they were able to bind inside the cavity via expansion of the cage (Figure 7a). In contrast, rupture of the cage to enable guest binding was observed by Yoshizawa and co-workers for binding of fullerene inside a [Hg_2_**L**_4_]^4+^ cage.^21^

Recognizing the dynamic nature of the binding process, recent efforts have shifted toward systems where guest binding is switched on/off, which has potential applications in drug delivery.^161^ Since the ligand–metal bond formation is in principle dynamic, the simplest way to alter binding is by using invasive methods based on full or partial disassembly of the metallo-organic cage. Nitschke and co-workers showed switchable binding of cyclohexane inside a [Fe_4_**L**^**n**^_6_]^4–^ tetrahedron (Figure 7b).^152^ This structure disassembles upon the addition of acid, presumably through the protonation and subsequent hydrolysis of the ligands. The process is then reversed by the addition of a base.

Crowley and co-workers have shown that the disassembly of a [Pd_2_**L**_4_]^4+^ cage, triggered by the addition of the strongly coordinating dimethylaminopyridine (DMAP), can be used to release the chemotherapeutic Cisplatin.^10^ The stronger basicity of DMAP compared to the cage ligand means that the cage reassembles when treated with *p-*toluenesulfonic acid (TsOH). It is interesting to compare the Crowley and Nitschke methods and the orthogonal way in which acid and base can be used to trigger both disassembly and assembly of cages. More recently, Crowley has applied the DMAP-responsive chemistry to a heteronuclear [PdPt**L**_4_]^4+^ cage. In this example, the DMAP selectively removes the more labile Pd(II) ion.^37^

Noninvasive stimuli such as light,^99,162^ temperature,^98^ or chemical signals^154,163,164^ have also been used to open cages reversibly. For example, Fujita and co-workers functionalized a [Pd_12_**L**_24_]^24+^ cage with internalized photoswitchable azobenzene units.^162^ Upon irradiation, the azobenzene group changed from *trans* to *cis* conformation, increasing the size of the cavity and allowing a hydrophobic guest, pyrene, to enter. This process could be reversed by heating. Clever and co-workers also inserted a photoswitch unit in the ligand of a [Pd_2_**L**_4_]^4+^ cage, resulting in contraction and expansion of the cage upon irradiation with light.^99^ The contracted structure was found to have the optimal size to bind [B_12_F_12_]^2–^.

### 4.2 Computational Prediction of Binding

Predicting host–guest binding is one of the central goals of computational chemistry.^165^ As a result, several methods have been developed, which differ in their accuracy and computational cost. For example, efficient but low-accuracy methods such as docking allow screening of a large number (∼10^9^) of possible guests.^166−168^ Docking relies on sampling possible binding modes that are subsequently ranked by a scoring function, which can be empirical, knowledge-based, or force-field-based. Related approaches include linear interaction energy (LIE)^169^ and molecular mechanics Poisson–Boltzmann/generalized Born surface area (MM-PBSA/MM-GBSA),^170−172^ which also include conformational sampling from short MD simulations and the implicit or explicit consideration of solvent. On the other hand, accurate methods include free energy perturbation (FEP),^173^ umbrella sampling (US),^174^ and metadynamics,^147,148^ which are based on extensive MD simulations of the unbound guest and host, and bound guest–host complex in explicit solvent. Although these methods have relatively high accuracy (<2 kcal mol^–1^ error),^79^ their computational cost makes them unsuitable for screening.

**Figure 8:**
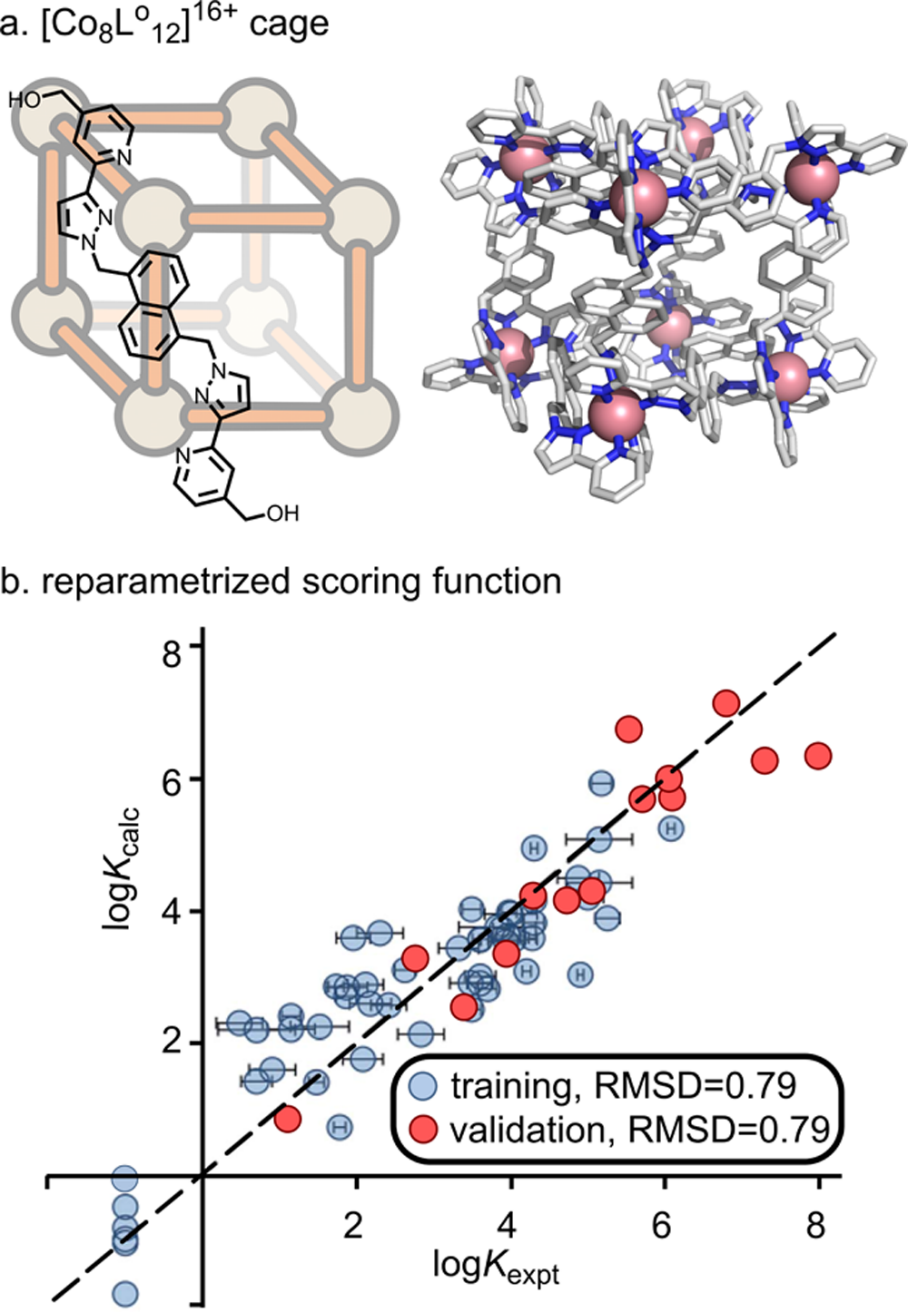
Docking of guests inside [Co_**8**_**L°**_12_]^16+^.^176^ (a) [Co_8_**L°**_12_]^16+^ cage structure. (b) Correlation between computed and experimental binding affinity (blue dots). The function was used to predict the binding of 3000 substrates, from which 15 were synthesized and their binding affinity calculated (red dots). Adapted from ref (176) with permission from the Royal Society of Chemistry.

Ward, Hunter, and co-workers employed the GOLD (Genetic Optimization for Ligand Docking) package^175^ to predict the binding affinity of 54 guests inside the [Co_8_**L**°_12_]^16+^ cage for which experimental data was available (Figure 8a).^176^ Initially, they employed the scoring function design for protein–ligand interaction, CHEMPLP;^177^ however, this resulted in a poor correlation to experiments (*R*^2^ = 0.02). A significant improvement was achieved by reparameterizing the scoring function to predict the association constant (log *K*) directly. The modified scoring function included only four parameters (ligand_clash, ligand_torsion, nonpolar, and buried part, RMSD = 1.0 kcal mol^–1^). Further improvement, especially for flexible guests, was obtained by including the number of rotatable bonds as a fifth parameter (RMSD = 0.5 kcal mol^–1^; Figure 8b). Using this function, they screened 3000 potential binders, from which 15 were experimentally characterized. An excellent agreement between computed and experimental binding affinity was obtained (RMSD = 0.5 kcal mol^–1^). Moreover, they found a new guest with a much higher binding affinity than previously reported, demonstrating that correct docking parametrization for a specific cage–guest system can aid the identification of strongly interacting substrates.

### 4.3 Computational Mechanistic Binding Studies

Complementary to experimental characterization, detailed computational studies have enabled the study of the mechanisms of guest encapsulation and release and the flexibility of the cage during these processes. Such studies have been performed, for example, to study the binding of fullerenes inside [Pd_4_**L**_8_]^8+^,^178−180^ charged guests inside [Ga_4_**L**^**p**^_6_]^12–^,^153^ photoswitchable [Pd_2_**L**_4_]^4+^ cages,^181^ and photoswitchable guests inside the [Pd_6_**L**_4_]^12+^ cage.^182^ Since binding occurs on the millisecond to second time scale, which is practically impossible to reach using conventional MD simulations, rare event sampling methods such as US,^174^ accelerated MD (aMD),^183^ the attach–pull–release (APR) method,^184^ and metadynamics^147,148^ are employed.

For example, Ribas and co-workers studied the binding of fullerenes inside [Pd_4_**L**_8_]^8+^ cages using a combination of ^1^H–^1^H exchange spectroscopy (2D-EXSY) NMR, conventional MD, and aMD with explicit solvent.^180^ The mechanism of fullerene binding was found to be regulated by the aromatic rings of cage ligands, which act as gatekeepers. The rate of the guest entrance was determined by the rotation of aromatic rings along the ligand axis.

**Figure 9:**
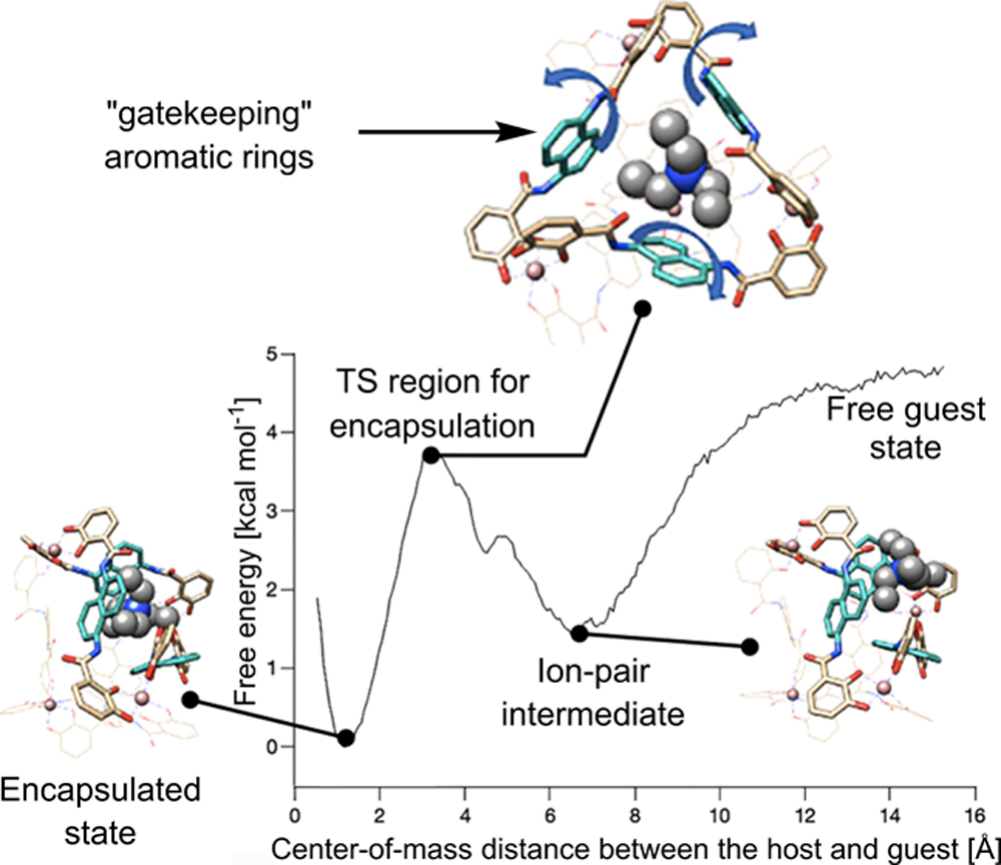
Computational mechanistic studies of binding. Binding pathway of NEt_4_^+^ inside the [Ga_4_L^p^_6_]^12–^ cage. Adapted from ref (153) with permission from the American Chemical Society.

Ujaque and co-workers used the APR method to study the binding of cationic guests inside [Ga_4_**L**^**p**^_6_]^12-^ (Figure 9). Their computed binding affinities were obtained within 2.3 kcal mol^–1^ of those obtained by NMR. Similar to Ribas and co-workers, they observed that the rotation of aromatic rings of the cage’s ligands controls the guest’s entrance, becoming the rate-limiting step for binding. Moreover, they found that the binding affinity strongly depends on the method of parametrization of the cage. For instance, using implicit solvation to calculate bonded parameters and partial charges significantly improved the results. Moreover, the use of low-cost metrics, such as the relative guest/cavity volume or guest volume, correlated with the binding affinities (*R*^2^ = 0.97 and *R*^2^ = 0.86, respectively). Despite the significant difference between sizes of guests (80–160 Å^3^), it was noticed that the 55% Rebek rule holds for guests if the encapsulated solvent molecules are considered.

Schäfer and co-workers have also studied the controllable uptake and release of [B_12_F_12_]^2–^ in a [Pd_2_**L**_4_]^4+^ cage with photoswitchable ligands.^181^ They calculated binding affinity using US with explicit solvent, which compared well to the value obtained from experiments (computed −6.7 kcal mol^–1^ vs −6.4 and −5.9 kcal mol^–1^ from ITC and NMR, respectively). Moreover, they estimated activation barriers of the removal of the guest from the cage with open, closed, or mixed open/closed ligands. When all the ligands are in a closed configuration, the binding affinity and activation barrier lower significantly, suggesting that the guest is released after closure of the first ligand of the cage.

Finally, Pavan and co-workers studied the binding and the *cis–trans* isomerization of azobenzene inside a flexible [Pd_6_**L**_4_]^12+^ cage.^182^ They performed MD simulations in explicit water solvent using a covalently bound metal model. Metadynamics simulations were used to calculate binding and kinetic parameters (*k*_on_ and *k*_off_). Moreover, the *trans–cis* isomerization time (τ_*trans–cis*_ = 1/*k*_*trans–cis*_) of azobenzene inside the cage was found to be orders of magnitude shorter than the residence time of the encapsulated guest (τ_off_), suggesting that isomerization occurs inside the cage. These examples demonstrate the unique opportunities that molecular modeling provides to gain insights into the mechanisms driving binding, which could be used for further optimization.

## 5 Catalytic Activity

### 5.1 Brief Summary of Experimental Studies

Mimicking enzymes by utilizing the defined cavities of supramolecular systems has been of significant interest to supramolecular chemists for many decades.^1,185^ Prominent catalytic coordination cages include the following: the [Ga_4_L^p^_6_]^12–^ cage, originally developed by Raymond and studied in collaboration with Bergman and Toste, which has been shown to catalyze a broad range of reactions, including aza-Cope^186−189^ and Prins rearrangements,^190^ Nazarov cyclization,^191−194^ hydrolysis of acid-labile compounds under basic conditions,^42,195,196^ alkyl–alkyl reductive elimination,^197,198^ the octahedral and bowl-shaped [Pd_6_L_4_]^12+^ cages developed by Fujita and co-workers, and other examples by Mukherjee,^199^ which have been shown to catalyze Diels–Alder^200−203^ and Knoevenagel reactions;^204^ and the octanuclear cubic [Co_8_L°_12_]^16+^ cages by Ward and co-workers, which have been shown to promote the Kemp elimination,^205^ phosphate ester hydrolysis,^206^ and aldol reactions^207^ (although the latter two examples have been shown to occur on the cage surface rather than inside). Recently, we demonstrated the catalytic activity of the [Pd_2_L_4_]^4+^ topology in Diels–Alder,^45^ Michael addition,^208^ and radical–cation cycloaddition reactions.^209^ For comprehensive reviews focused on the experimental studies of cages catalysis, we refer to the relevant literature.^30,210−212^ While these promising examples demonstrate the potential of metallo-organic cages to achieve selectivity and activity not possible with other synthetic catalysts, the limited number of examples show that the design of these systems is challenging.

In the examples reported to date, acceleration occurs either because of enthalpic stabilization, i.e., efficient and selective recognition of intermediates and TS, versus the reactants and products, or via entropic mechanisms, i.e., increasing the effective concentration of reactants, or by constricting acyclic substrates. However, for catalysis to occur it also requires turnover; therefore, the relative association constants for the reactants and products are key. Usually, all these requirements may be difficult to balance.

### 5.2 Computational Cage Catalysis

Computational modeling provides atomic-level insight into the fundamental aspects of cage catalysis, helping to rationalize experimental observables and predict possible outcomes.^213^ From these investigations, several factors have been identified as crucial for catalytic activity, including reduction of entropy, (relative) destabilization of reactant complexes, TS stabilization, distortion, and microsolvatation. In the next paragraphs, we discuss relevant examples where computation has helped elucidate the origin of metallo-organic cage catalysis. We will not cover computational studies on supramolecular capsules (organic noncovalent cages) and refer the reader to relevant works in this area.^213−216^

#### 5.2.1 Gallium [Ga_4_L_6_]^12–^ Cage

##### Orthoformate Hydrolysis

**Figure 10:**
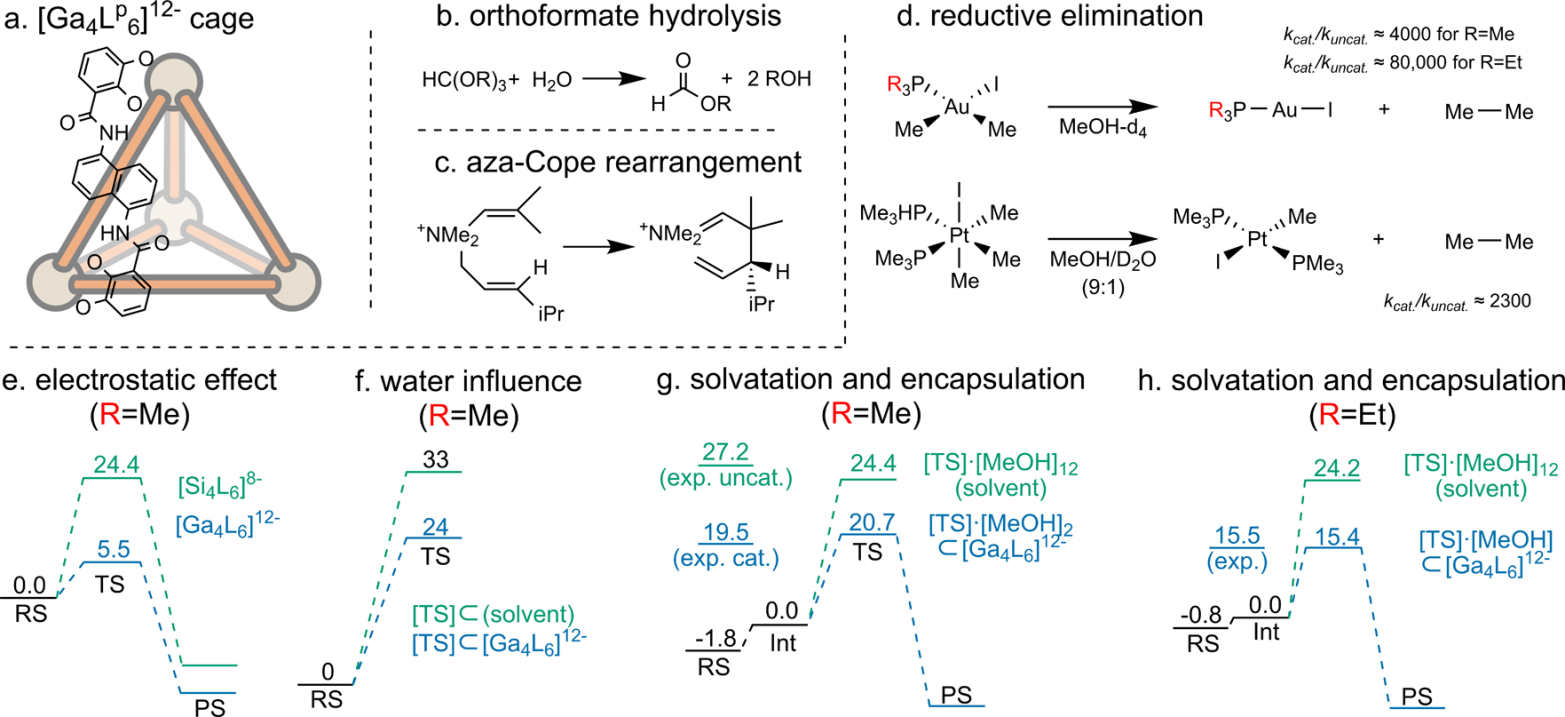
Catalysis in the [Ga_4_**L**^**p**^_6_]^12–^cage. (a–d) Structure and reactions catalyzed by the cage.^197^ (e–h) Computational studies on the origin of catalysis for reductive elimination: (e) electrostatic effect,^197^ (f) solvation effects inside and outside the cage,^219^ (g) explicit solvatation and encapsulation,^221^ and (h) catalysis of triethyl-substituted complex.^222^

The cage-catalyzed hydrolysis of orthoformates and acetals was reported by Raymond and co-workers, who showed that the [Ga_4_**L**^**p**^_6_]^12–^ cage promotes these reactions at high pH, whereas the bulk-phase reaction only occurs under acidic conditions.^42^ Warshel and co-workers were the first to computationally study the mechanism of this reaction inside the [Ga_4_**L**^**p**^_6_]^12–^ cage (Figure 10a).^217^ They employed the EVB approach,^81^ which was parametrized to fit the reference hydrolysis of two orthoformates in water. These parameters were used unchanged to model the reaction inside the cage (Figure 10b).^217^ While TS stabilization was found to be important, the overall catalytic activity was found to primarily arise from electrostatic preorganization of the H_3_O^+^ species, leading to a very low “local pH” inside the cage even when the external solution was kept at a high pH.

##### Aza-Cope Rearrangement

The cage-catalyzed 3-aza-Cope rearrangement of allyl enammonium cations to iminium cations (which subsequently hydrolyze to the corresponding aldehydes) was also reported by Raymond and co-workers. The reaction was accelerated by a factor of 850 inside the [Ga_4_**L**^**p**^_6_]^12^ cage (Figure 10c).^187,189^ The origin of this effect and the selectivity preference for the *R*-enantiomer were computationally investigated by Nakajima and co-workers.^218^ They performed QM/MM calculations, with the cage and substrate modeled at the QM (B97D/def2-SV(P) and MP2/def2-SV(P)//B97D/def2-SV(P)) levels of theory and the 12 countercations in the MM region. The effect of solvent, modeled implicitly, was found to be negligible. The authors computed enthalpy only as it was experimentally shown that entropy reduction played a major role in catalysis but not in enantioselectivity. The computed enthalpies of activation, Δ*H*^‡^, for the uncatalyzed and catalyzed reactions were in excellent agreement with experimental values; uncatalyzed comp. 24.5 kcal mol^–1^ vs exp. 23.6 kcal mol^–1^ and catalyzed 21.7 kcal mol^–1^, exp. 22.7 kcal mol^–1^. The preference for the *R*-product was suggested to originate from the different stability of the prochiral structures inside the cage due to deformation of the bulky substituent.

##### Reductive Elimination

In 2015, Toste and co-workers reported that the C–C reductive elimination reaction of high-valent [Au(III), Pt(IV)] metal alkyl complexes is accelerated inside [Ga_4_**L**_6_]^12–^ cages (Figure 10d).^197^ This elementary reaction was incorporated into a dual cross-coupling cycle, for which the metal complex and supramolecular cage were required for efficient turnover (TON > 300). The C–C bond-forming reaction was suggested to proceed via a pre-equilibrium halide dissociation followed by a transient and reversible encapsulation of the nascent organometallic cationic species, which then undergoes irreversible elimination inside the cavity. In their subsequent studies, they evaluated the effect of spectator ligands, reactive alkyl ligands, solvent, and catalyst structure.^198^ In particular, they observed an increased reaction rate upon increasing the water content of the methanol solvent and by substituting the methyl phosphine ligand for ethyl.

The first computational rationalization of this [Ga_4_**L**^**p**^_6_]^12–^ catalyzed reaction was reported by Head-Gordon and co-workers using DFT (ωB97X-v/TZV2P(Au),DZVP).^219^ They hypothesized that the negatively charged cage stabilizes the positively charged intermediate and TS (Figure 10e). Indeed, the activation energy for the reductive elimination step was found to be lower for the charged intermediate than for the uncharged substrate. Electrostatic stabilization was further confirmed by comparing this cage to a lesser charged analogue ([Si_4_**L**^**p**^_6_]^8–^). Moreover, the high turnover of the catalyst was rationalized based on the stronger binding of the reactant state compared to the product. Their study suggested that the addition of water would decrease the activation energy; however, this effect was not quantified.

In a subsequent study, they investigated the role of water using ab initio MD (AIMD; B97M-rV/DZVP) simulations in an explicit water solvent (Figure 10f). The calculated acceleration rate was in reasonable agreement with the experiment (comp. 3.3 × 10^7^ vs exp. 5.0 × 10^5^–2.5 × 10^6^). Electrostatic effects and the presence of a single complexed water molecule inside the cage, which organizes the electrostatic potential, were suggested to facilitate the reaction. They also noted that, unlike enzymes, the cage poorly reorganizes water on its surface. Since they found that the unorganized interfacial water is detrimental for TS stabilization, it was suggested that even better catalytic acceleration could be achieved by enhancing the ordering of the solvent inside the cage. They suggested that substitution of gallium for indium ion would reduce the energy required for solvent reorganization and result in stabilization of the transition state.^220^

The same reaction has been studied by Ujaque and co-workers using MD simulation with methanol explicit solvent and DFT (SMD-B3LYP-D3/6-31G(d),SDD(Au)). MD simulations were used to evaluate the number of solvent molecules inside the cage while DFT was used to study catalytic activity (Figure 10g).^221^ On average, two methanol molecules were found to be present inside the cage. The activation barrier for the uncatalyzed reaction was calculated with DFT using implicit solvent and 12 explicit methanol molecules. For the reaction in the cage, two explicit methanol molecules were placed inside the cavity. The computed barriers were in excellent agreement with the experimental values (uncatalyzed: exp. 27.2 kcal mol^–1^ vs comp. 26.2 kcal mol^–1^ and cage-catalyzed: exp. 20.7 kcal mol^–1^ vs comp. 19.5 kcal mol^–1^). Using an energy decomposition analysis, they identified that TS stabilization arises from its encapsulation and interaction with the cage (3 kcal mol^–1^). Additionally, in solution, the formation of the charged intermediate requires dissociation of iodide from the saturated organometallic complex, which is slightly endergonic (Δ*G* = 1.8 kcal mol^–1^). They investigated the role of the solvent by removing explicit methanol molecules from the uncatalyzed reaction. Removal of the 10 methanol molecules, leaving only two, had a minor effect (0.7 kcal mol^–1^) on the activation barrier. However, removal of the remaining two molecules significantly decreased the activation barrier by 7 kcal mol^–1^. Since simulations show two methanol molecules inside the cage, it was proposed that the solvent has a negligible effect on catalysis. However, the authors noted that hypothetical removal of the remaining methanol molecules from the cage would significantly lower the activation barrier.

In a different study, Ujaque et al. also investigated the effect of changing the substrate’s trimethyl phosphine ligand to triethyl (Figure 10h).^222^ As expected, a larger ligand led to less solvent encapsulated by the cage. Their MD studies showed that in equilibrium, the cavity is occupied by 5–8 methanol molecules and surrounded by 6–9 K^+^ ions with up to two inside the cavity. As a result, the cage has an effective charge in the range −6 to −3. Upon encapsulation of the charged Au(III) iodide complex, the iodide rapidly leaves the cage, and only one methanol stays inside.

They also investigated the reaction in a vacuum and explicit solvent, modeling the cage using DFT (SMD-B3LYP-D3/6-31G(d),SDD(Au)). Compared to the uncatalyzed reaction with 12 explicit methanol molecules, they observed a decrease of 8.8 kcal mol^–1^ barrier for the reaction inside the cage. Overall, two factors were found to contribute to catalytic activity: desolvatation, with removal of 11 explicit methanol molecules (leaving only one in the complex) decreasing the activation energy by 5.7 kcal mol^–1^, and the electrostatic interaction between the TS and the cage, which reduces the barrier by 3.1 kcal mol^–1^. Surprisingly, the surrounding ions, which effectively increase charge, did not have a significant effect on the activation barrier.

They extended this analysis to the reductive elimination of a Pt(IV) complex.^223^ Despite the similarity of the Au(III) and Pt(IV) complex reactions, the factors contributing to catalytic activity differ. In the Pt(IV) complex, catalytic activity was found to arise from encapsulation effects (6.5 kcal mol^–1^) rather than microsolvatation (0.5 kcal mol^–1^) as was observed in the Au(III) complex.

#### 5.2.2 Pd-Cage Catalysis

##### Diels–Alder Reaction

**Figure 11:**
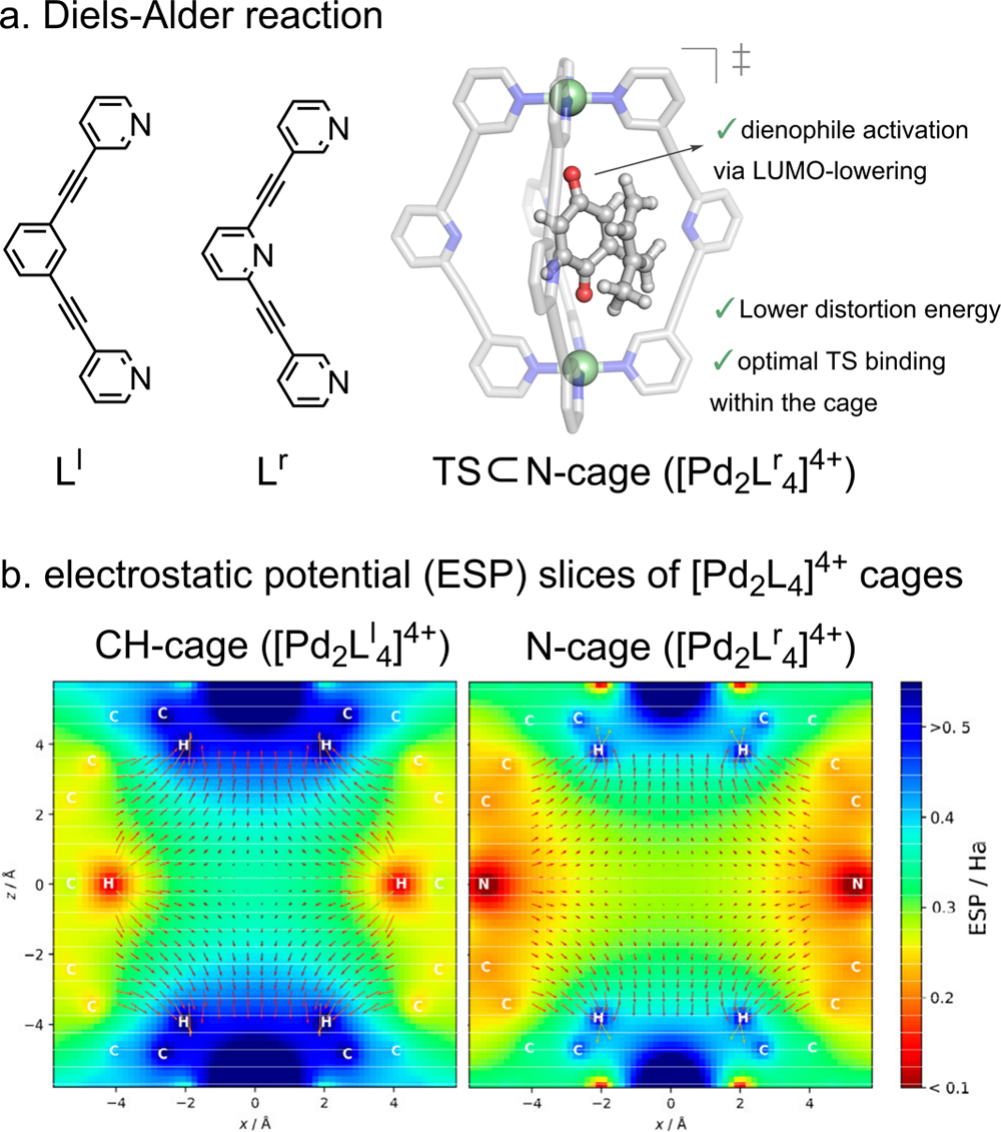
Reactivity in [Pd_*n*_**L**_2*n*_**]**^2*n*^^+^. (a) Diels–Alder reaction is catalyzed by the N-cage ([Pd_2_L^r^_4_]^4+^).^226^ (b) Electrostatic potential (ESP) slices of the CH-cage ([Pd_2_L^l^_4_]^4+^) and N-cage ([Pd_2_L^r^_4_]^4+^) on the *xz* plane containing two opposing ligands and two metal centers.^208^ Reprinted with permission from refs (208) and (226), from the American Chemical Society.

The [Pd_2_**L**_4_]^4+^ topology originally reported by Steel and McMorran,^224^ and later utilized by Clever,^59,61,99,101^ Hooley,^225^ and Crowley,^10^ has been extensively used for binding, transport, and catalysis. In 2018, we evaluated the ability of [Pd_2_**L**^**l**^_4_]^4+^ and [Pd_2_**L**^**r**^_4_]^4+^ cages to catalyze Diels–Alder (DA) reactions using quinone substrates as dienophiles. In these systems, the linkers differ in having either benzene or pyridine as central group, and therefore, they are referred to as CH-cage ([Pd_2_**L**^**l**^_4_]^4+^) and N-cage ([Pd_2_**L**^**r**^_4_]^4+^), respectively (Figure 11a).^45^ Unlike the cage-catalyzed reactions described above, which take place in water, this system operates in dichloromethane with noncompeting BArF^–^ anions, enabling enthalpic activation of the substrate via C–H hydrogen bond interactions. It should be noted that unlike previous examples of cage-promoted Diels–Alder reactions,^195−198^ this catalysis only involves formal binding of the dienophile, and so there is no contribution from increased effective concentration. While the N-cage was found to be catalytic, with rate accelerations (*k*_cat_/*k*_uncat_) of up to 10^3^, the CH-cage was inactive, despite the latter having strong substrate binding. The contrasting catalytic ability was postulated to arise from a combination of weakened substrate binding (ground-state destabilization) and enhanced TS stabilization.

To rationalize how those subtle structural differences in the cage framework affect binding and catalysis, a computational protocol was developed, employing MD and DFT methods.^226^ MD simulations in explicit dichloromethane (DCM) solvent were used to evaluate the flexibility of the cage, using a modified version of Yoneya’s Pd^2+^ dummy model (Section 3.2). Higher flexibility for the N-cage compared to the CH-cage was identified, suggesting that the active N-cage can accommodate significant deformation to accommodate the increasing bulk of the TS without the energetic penalty that would be expected for the CH-cage.

Guest binding was calculated using DFT with the SMD(DCM)-M06-2X/def2-TZVP//PBE0-D3BJ/def2-SVP level of theory, which previously was shown to accurately describe association energies for large supramolecular systems.^227^ Relative (ΔΔ*E*_bind_) rather than absolute binding energies were calculated. This avoids having to consider entropic contributions, which can introduce significant errors, as they are expected to be similar in both systems.^228,229^ The relative binding affinities of a range of quinone-based guests were calculated with very good accuracy (MAD = 1.9 kcal mol^–1^, *R*^2^ = 0.76).

Activation energies for the reaction between different benzoquinones and dienes were calculated at the SMD(DCM)-M06-2X/def2-TZVP//PBE0-D3BJ/def2-SVP level of theory. In addition, a distortion-interaction analysis was used to determine the components of the activation energy. A more favorable interaction energy than for the uncatalyzed reaction was observed for both cages, which arises from lowering of the dienophile LUMO energy and enhancement of its electrophilic character. However, the lack of catalytic activity in the CH-cage was found to arise from a large distortion penalty due to steric clashes between the ligand CH moieties and dienophile at the TS (Δ*E*^⧧^_cat_ > Δ*E*^⧧^_uncat_). To decrease the computing time when estimating catalytic proficiency, “TS analogues” were used, where bonds being formed and broken are constrained to the values found in the uncatalyzed TS. This strategy resulted in >80% accuracy in classifying catalytic cages and reduced the computational time by 10 times.

##### Base-free Michael Addition Reaction

In a subsequent study, the CH-cage was found to efficiently catalyze several Michael addition reactions, while the N-cage was catalytically inactive.^208^ The cage promoted the spontaneous, base-free pro-nucleophile deprotonation via stabilization of the conjugated anion, with an acidity enhancement comparable to several p*K*_a_ units. The calculated electrostatic potential of the two cages showed that the nitrogen atoms of the N-cage significantly neutralize the remote charge arising from the Pd^2+^ ions, making the central cavity much less electropositive than the CH-cage (Figure 11b). This means that the CH-cage is better at stabilizing anionic species compared to the N-cage, hence explaining the reactivity difference. The cage also promoted different levels of diastereoselectivity for reactions in which the product possesses multiple stereocenters. This effect has been probed with DFT calculations at the SMD(DCM)-M06-2X/def2-TZVP//PBE0-D3BJ/def2-SVP level of theory, which showed a pronounced bias toward encapsulating one of the diastereomers inside the cage, supporting the observed diastereoselectivity in the reactions.

## 6 Overview and Future Perspectives

In recent years, substantial progress has been made toward the design of structurally diverse metallo-organic cages. For example, simple geometric principles have been successfully employed to guide the design of increasingly complex systems. However, functional metallo-organic cages, for example, as catalysts, remain underdeveloped. Despite the hundreds of assemblies reported in the past three decades, catalysis appears limited to a few privileged structures.

By reviewing the processes enabling the functional activity of these systems, we have illustrated the key aspects that need to be considered to study metallo-organic cage design and move away from current trial-and-error approaches. While substantial challenges remain, we demonstrate that existing computational tools can already help interpret and, in some cases, predict experimental observations. For example, efficient open-source computational tools have enabled the screening of hundreds of new designs, saving precious time and resources for experimentalists. Ensuring that these tools continue to be developed and are accessible to experimentalists will be essential to generating a feedback loop between computational and supramolecular chemists.

Only in the past decade have techniques extensively used in enzyme modeling, such as QM and MD, been employed to study processes such as cage assembly, binding, and catalysis. Still to overcome are the difficulties related to the quality of the models and efficiency of the techniques available. For example, the choice of the force fields and inclusion or not of solvent and/or counterions have a tremendous influence on the results of self-assembly and binding simulations. Moreover, most classical force fields are intrinsically unsuitable for studying cage formation, as they lack charge transfer. Therefore, high-quality and easy to generate force fields that accurately describe charge transfer and polarization will be necessary. Recently developed machine-learned potentials could help bridge the current gap between classical and quantum techniques, providing the accuracy and efficiency required. The community will also need to go beyond conventional modeling techniques targeted at describing thermodynamic minima and introduce efficient enhanced sampling techniques able to describe processes that experimentally take place on the scale of seconds or beyond. Moreover, knowledge of the pathways involved will enable us to explore kinetic traps from where novel designs could arise.

Finally, in the realm of catalytic cage design, detailed mechanistic studies on targeted catalytic activity may still be necessary. This will require efficient and accurate electronic structure methods that enable the calculation of large QM regions and their accurate sampling. Advances in this area include linear scaling DFT approaches, the introduction of GPU architectures, and the development of improved semiempirical methods such as xTB. These studies could then be complemented with large-scale screening efforts and machine-learning techniques to further explore the available chemical space. Collaboration with experimentalists is key to success, which will give access to both positive and negative results, such that the quality of the models and accuracy of the conclusions will be better estimated.

Computational chemistry shows great promise for the design of supramolecular cages. We envision that the pioneering efforts highlighted in this review will be expanded to create more robust and efficient design methods. The synergy between rapid screening approaches, accurate molecular modeling, and experimental validation will enable us to go beyond traditional approaches of intuition-driven trial-and-error, reducing the overall time and cost needed to discover new functional cages.
